# A systematic map of methods for assessing societal benefits of Earth science information

**DOI:** 10.1073/pnas.2524370123

**Published:** 2026-02-06

**Authors:** Casey C. O’Hara, Mabel Baez-Schon, Rebecca Chaplin-Kramer, Samantha H. Cheng, Alejandra Echeverri, Gillian L. Galford, Rachelle K. Gould, Cristina L. Mancilla, Maura C. Muldoon, Gerald G. Singh, Priscilla Baltezar, Yusuke Kuwayama, Stephen Polasky, Amanda D. Rodewald, Richard P. Sharp, Elizabeth J. Tennant, Jiaying Zhao, Benjamin S. Halpern

**Affiliations:** ^a^National Center for Ecological Analysis and Synthesis, University of California, Santa Barbara CA 93101; ^b^Global Science, World Wildlife Fund, Washington, DC 10024; ^c^Department of Environmental Science, Policy and Management. University of California, Berkeley, CA 94709; ^d^Rubenstein School of Environment and Natural Resources, University of Vermont, Burlington VT 05401; ^e^Gund Institute for Environment, University of Vermont, Burlington VT 05401; ^f^School of Environmental Studies, University of Victoria, Victoria BC V8P 5C2, Canada; ^g^Space Enabled Research Group, Massachusetts Institute of Technology, Cambridge MA 02139; ^h^School of Public Policy, University of Maryland, Baltimore, MD 21250; ^i^Department of Applied Economics, University of Minnesota, St. Paul MN 55108; ^j^Cornell Lab of Ornithology and Department of Natural Resources and the Environment, Cornell University, Ithaca NY 14850; ^k^Charles H. Dyson School, Cornell University, Ithaca NY 14853; ^l^Department of Psychology, Institute for Resources, Environment and Sustainability, University of British Columbia, Vancouver BC V6Z 1T4, Canada; ^m^Bren School of Environmental Science and Management, University of California, Santa Barbara CA 93106

**Keywords:** satellite, remote sensing, value of information, societal benefit, Earth observation

## Abstract

Remotely sensed Earth science information (ESI) has become increasingly central to addressing global challenges, yet its societal value, i.e., the difference ESI makes in real-world decisions and outcomes, is rarely quantified. In this study, we systematically map peer-reviewed literature that explicitly assesses the societal value of ESI across instrumental, intrinsic, and relational value types, and the diversity of approaches used to assess those values. Drawing from 13,823 publications across Scopus, Web of Science, and a curated library of ESI valuation studies, we identify 171 studies that applied ESI in a decision context and used a valuation method to compare outcomes with and without ESI. The majority of these studies employed decision analysis methods (e.g., Value of Information, Cost–Benefit Analysis), focusing primarily on quantitative instrumental values (e.g., profit, crop yield, lives saved), particularly in agricultural contexts. A smaller set of studies applied preference elicitation methods (e.g., stated preference, surveys, interviews, focus groups) to capture qualitative benefits and relational values including quality of life improvements, empowerment, and procedural justice. Many excluded studies demonstrated scientific value of ESI but did not explicitly translate that into societal value, revealing the need for a more systematic approach to ESI valuation. By promoting a more inclusive, interdisciplinary, and flexible portfolio of valuation methods, we aim to expand our understanding of the societal benefits of ESI to help guide investment in future missions, enhance public support, and ensure that science and policy goals are well aligned.

## Significance

Earth science information (ESI) from satellites and other remote sensing technologies is critical for managing climate, agriculture, disasters, and more. Yet the societal value of ESI, how it improves real-world decisions and outcomes, remains poorly understood. We systematically map studies that quantify this value, revealing how different methods capture diverse benefits, from economic efficiency and lives saved to empowerment and justice. Our findings demonstrate that a rich array of methods exists to assess societal benefits of ESI across many decision contexts, identifying benefits in terms of instrumental and relational values. This synthesis expands the evidence base for why ESI matters and how it can help guide future investments, promote public support, and align Earth science with societal goals.

In recent decades, remotely sensed information about the state of our planet has become increasingly vital for understanding and addressing global challenges such as climate change, water resource management, biodiversity conservation, sustainable development, and public health (1–4). Rapid technological progress has led to widespread availability of Earth observation data comprising global or regional datasets from remote sensing (e.g., satellite hyperspectral imagery, aerial drone photography, in-situ terrestrial and marine sensor networks), at increasingly detailed and nearly continuous spatial and temporal coverage of the planet’s surface (5). These data are transformed, through algorithms, analyses, and models, into Earth science information (ESI) products such as land cover maps, climate forecasts, and drought early warning systems that managers and policy makers can use to inform societally consequential decisions (6). Advances in the availability and sophistication of ESI have accelerated its application across a wide range of decision contexts (7), supporting societal benefits as varied as impeding transmission of polio in Nigeria (8), protecting blue whales in the Eastern Pacific (9), improving targeting of cash transfers to poor villages in sub-Saharan Africa (10, 11), and empowering Indigenous communities to monitor deforestation (12). While the contributions of ESI to such societal benefits are undeniable, the magnitude and diversity of these contributions are rarely assessed, and the requisite conditions for success are rarely examined. Yet if we do not understand the value of ESI, we risk underinvesting in information essential for protecting or enhancing our quality of life.

The gap in valuation of ESI is due in part to the separation of Earth system science from social and decision sciences, and further compounded by a lack of information-valuation frameworks that integrate diverse values. Understanding the breadth and magnitude of societal benefit of ESI, i.e., the value of practical application of data and data products toward socially desirable outcomes that goes beyond scientific merit (2), is important for guiding development of information that is actionable, meaningful, and credible for society’s needs, thereby justifying investment in future missions, garnering public support, fostering ESI uptake, and ensuring that science and policy goals are well aligned.

Inclusive and pluralistic value systems have long been a topic of discussion in conservation science and sustainable development (e.g., 13–16). The Intergovernmental Science-Policy Platform on Biodiversity and Ecosystem Services (IPBES) Values Assessment (17), a multiyear effort by scores of experts in diverse forms of valuation, identified three categories of value that reflect the ways in which nature and ecosystems are important for people: instrumental value as a means to satisfying specific human needs or interests, e.g., more revenue, higher crop yield, better health outcomes; intrinsic value independent of reference to people as valuers, e.g., the right of a whale to exist without regards to human preferences; and relational value deriving from meaningful and often reciprocal human relationships beyond means to an end, e.g., connection with a sacred landscape, a sense of responsibility toward one’s community (18) (*SI Appendix*, Table S1). Failing to capture potential gains related to noninstrumental values will greatly underestimate the contribution of ESI to societal benefits.

The mechanism by which ESI, indeed any information, generates societal value is through its ability to improve decision making toward socially desirable outcomes. It does this by aiding in the scoping of decision contexts and in the assessment of alternatives, thus reducing the likelihood of making decisions that result in unforeseen or undesirable outcomes. Economic frameworks to quantify the “value of information” typically calculate the difference in expected outcome of a decision made in a world with, versus without, the information (e.g., 19). Such decision analysis methods have played a critical role in demonstrating ESI’s potential to improve societal outcomes measured in instrumental values (i.e., means to an end, such as improved profits or crop yields) (20, 21). Value of information models based on decision analysis are well suited to quantifying socially desirable outcomes in terms of instrumental value given specific assumptions around the decision context and statistical parameters, but these models are not well-suited to capture the qualitative ways in which ESI can contribute to intrinsic and relational values such as fair decision processes, sustainability, justice, and human well-being (22). Other valuation methods can account for instrumental and noninstrumental values alike by eliciting individual and societal preferences for goods and services through quantitative, qualitative, and mixed-methods approaches (e.g., market price, stated/revealed preference, surveys, interviews, focus groups) (23). While these methods are commonly used for valuation of goods and services, they also bear potential for valuation of information when they are used to account for the value of the differences between a decision made with ESI relative to the decision made without. However, the extent to which these various methods have been successfully applied to translate the scientific value of ESI into explicit societal value, across decision contexts and types of societal values, remains poorly documented.

Here we present a systematic map of the peer-reviewed literature to identify studies in which a valuation method was used to compare the result of a decision supported by ESI. In this study, we aim to address four questions: 1) To what degree have various valuation methods been used to evaluate the societal value of ESI? 2) Which types of value (instrumental, intrinsic, relational) do these methods capture, and how are these values articulated? 3) How is the application of various valuation methods distributed across sectors and decision contexts? And finally, 4) what opportunities exist to develop more inclusive, systematic, and interdisciplinary approaches to ESI valuation? Here we present a systematic map of the peer-reviewed literature to identify studies in which a valuation method was used to compare the result of a decision supported by ESI to the result supported by some alternate information source. Through this systematic map of the peer-reviewed literature across a wide range of disciplines, we characterize the current landscape of ESI valuation, identify methodological and disciplinary gaps, highlight emerging practices, and point toward a more pluralistic and actionable valuation framework. Understanding and improving the way we value information will promote investments in Earth observations that not only expand scientific understanding but also deliver equitable and measurable benefits across diverse communities and decision contexts.

## Results

### Screening Process.

**Fig. 1.**
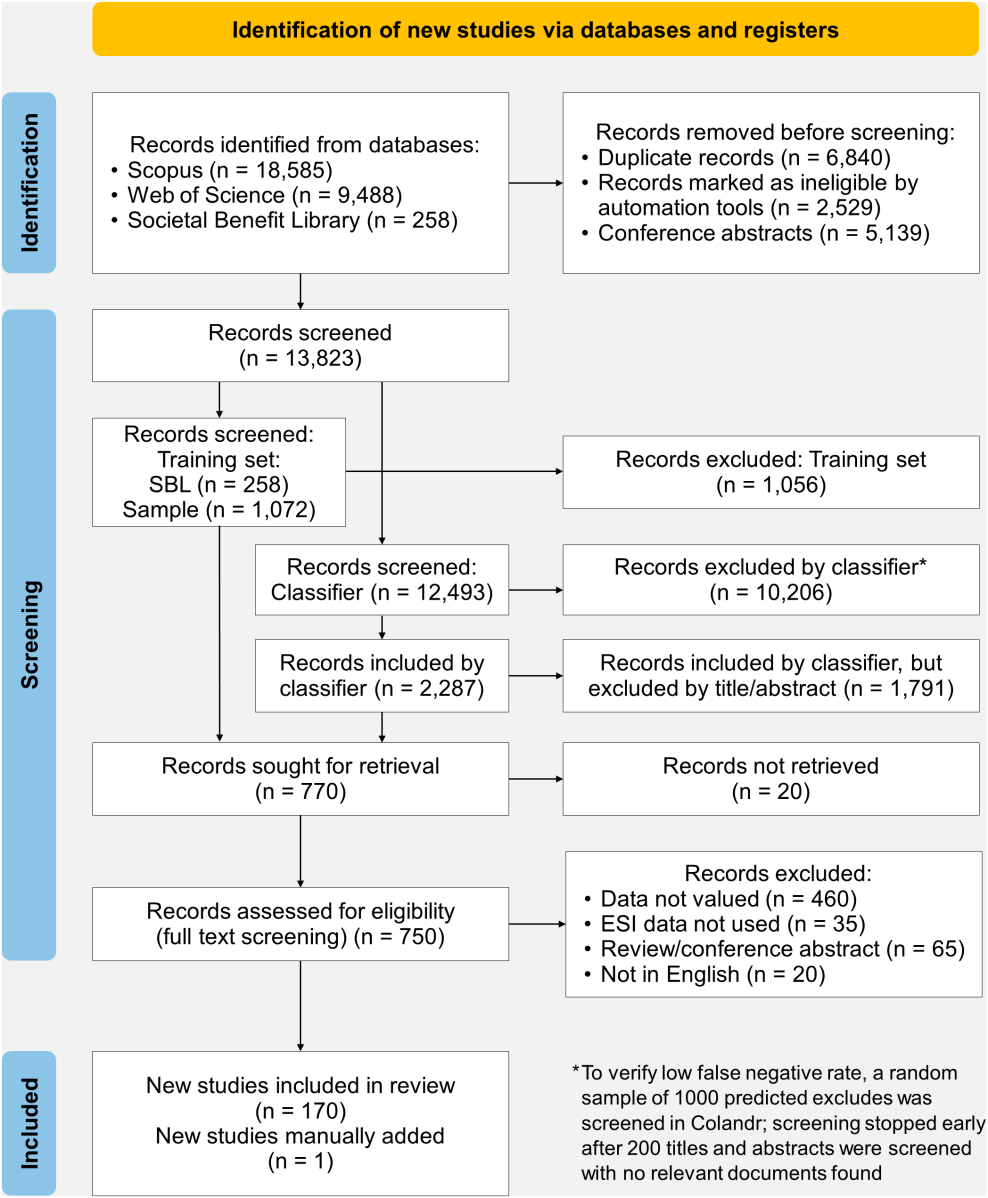
Citation identification, prescreening, and screening results. Following prescreening, n = 13,823 documents proceeded to title/abstract screening stage. A set of 1,330 documents was fully screened and used to train a machine learning classifier model, which was used to predict the status of the remaining 12,493 documents. Following full text screening, n = 170 documents were included in our analysis. Based on expert knowledge, one additional document was added manually, resulting in a final corpus of n = 171 documents.

Application of the search string (*SI Appendix*, *Methods* and Fig. S1) to Scopus and Web of Science databases, combined with references from an existing curated library of ESI valuation literature (Societal Benefits Library, SBL) (not available online at time of publication), yielded 28,331 records. Prescreening eliminated conference abstracts, spurious matches, duplicates, and incomplete records, narrowing the corpus to 13,823 unique citations (Fig. 1). Unique citations were then assessed for inclusion according to the following criteria: 1) the study made substantive use of ESI; 2) ESI was applied in a decision context; 3) the predicted or realized outcome of an ESI-based decision was compared to that under an alternative information set; and 4) the difference in outcome was presented in terms of some societal benefit. Reviews were excluded as they do not present original data. After prescreening, the complete records in the SBL (n = 258) and an additional 1,072 randomly sampled documents were manually screened by the team and used as a training set to inform a machine-learning classifier model on the remaining 12,493 citations. This classifier model predicted 2,287 documents as “includes” and the remaining 10,206 documents as “excludes.” All predicted “includes” then went through the same title/abstract screening process as the SBL and training sample, to eliminate false positives. A subset of the predicted “excludes” (n = 1000) was also screened to verify a negligible false negative rate. Of the 13,823 unique citations, 770 documents sufficiently met the title/abstract screening criteria, and full texts were subjected to final screening. Full text screening resulted in 170 documents that met all criteria (Fig. 1), to which one more document was added based on prior knowledge of a coauthor. All title/abstract screening was performed in the Colandr web-based machine-learning assisted screening app (24). Zotero reference management software was used to retrieve and organize documents for full text screening. The final list of 171 documents included in our study is listed in the supporting information (*SI Appendix*, Table S3).

### Valuation Methods.

**Fig. 2.**
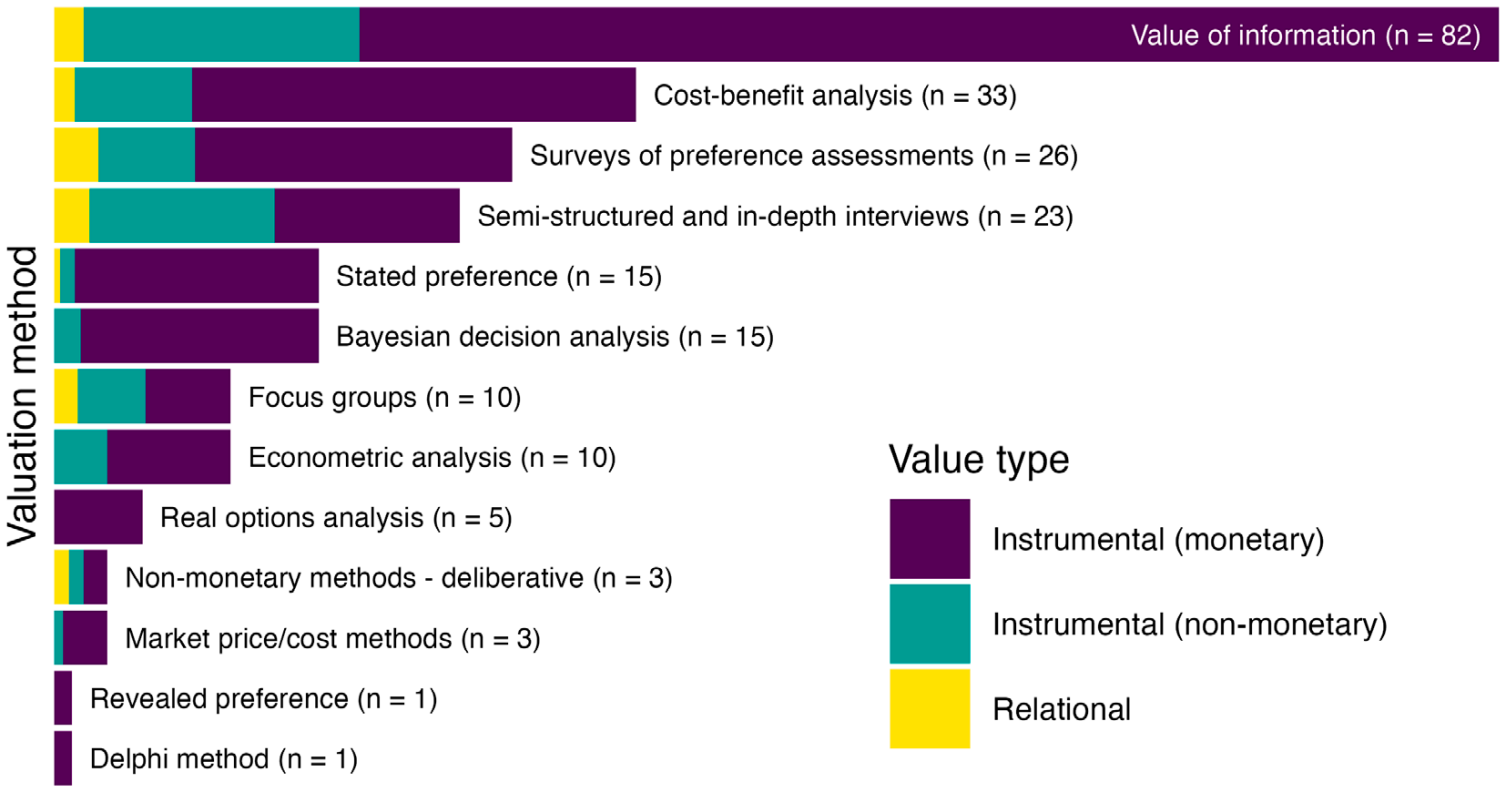
Number of valuation methods observed across included studies. Some studies applied multiple methods for valuation, thus the total number of observed methods (227) exceeds the total number of included studies (171). Color indicates value types assessed: instrumental, i.e., means to an end, including both monetary and nonmonetary outcomes, and relational, i.e., deriving from meaningful and often reciprocal relationships among people, nature, and society (*SI Appendix*, Table S1). We found no studies that measured benefits in terms of intrinsic value.

To examine the degree to which various methods have been applied to assess the societal benefits of ESI, we examined the full text of each of the 171 included papers and noted the primary valuation method (and where applicable, a secondary method) as implemented in the study (*SI Appendix*, Table S2 for operational definitions used to categorize valuation methods, *SI Appendix*, Table S4 for tabulation of valuation methods, decision contexts, and value types). The most common approaches we identified in the literature were quantitative economic approaches grounded in decision analysis: Value of Information (VOI) framework (n = 82; 48% of 171 included studies) and Cost–Benefit Analysis (CBA) (n = 33; 19%) (Fig. 2). Applied qualitative or subjective methods were also frequently observed, including surveys of preference assessments (n = 26; 15%) and semistructured or in-depth interviews (n = 23; 13%). Deliberative and consensus-based approaches were rare (n = 3 and 1 studies, respectively). Methods based on decision analysis (n = 145) were more frequently observed than methods based on preference elicitation (n = 82) (See *SI Appendix*, Table S2 for categorization of methods as decision analysis vs. preference elicitation). Nearly a third (56 studies) of the 171 included documents applied more than one information valuation method, with a total of 227 instances of valuation methods being observed (Fig. 2).

To understand the distribution of value types assessed in the ESI valuation literature, we coded each study to indicate whether the societal benefit was expressed in terms of monetary instrumental value (e.g., improved profit), nonmonetary instrumental value (e.g., pollution reduction, lives saved), intrinsic value (e.g., the moral right of nonhuman species to exist in peace), and/or relational value (e.g., connection with land, poverty alleviation, social justice, knowledge transfer among community) (see *SI Appendix*, Table S1 for definitions and examples of value types, and *SI Appendix*, Table S4 for tabulation of methods, contexts, and value types across all studies). Included studies most commonly measured societal benefits in terms of monetary instrumental value (n = 154) and/or nonmonetary instrumental value (n = 63), with many studies accounting for multiple instrumental metrics simultaneously (e.g., reduced crop pesticide application and the associated increase in profit; see *SI Appendix*, Table S1 for definitions and examples). Studies that measured societal benefits in terms of relational value were far less common in the literature (n = 17) and were more frequently assessed using qualitative preference elicitation methods, especially surveys, interviews, and focus groups. Studies rarely focused exclusively on outcomes associated with relational value, but typically examined relational value alongside instrumental value; for example, recreational fishing both as a pastime and as an economic activity (25). No papers in our study described decision outcomes in intrinsic terms; one paper (9) examined potential for ESI to inform regulation to reduce fatal ship strikes of blue whales, but the researchers focused on compliance with conservation policy, an instrumental goal, rather than any intrinsic rights of the whales themselves.

**Fig. 3.**
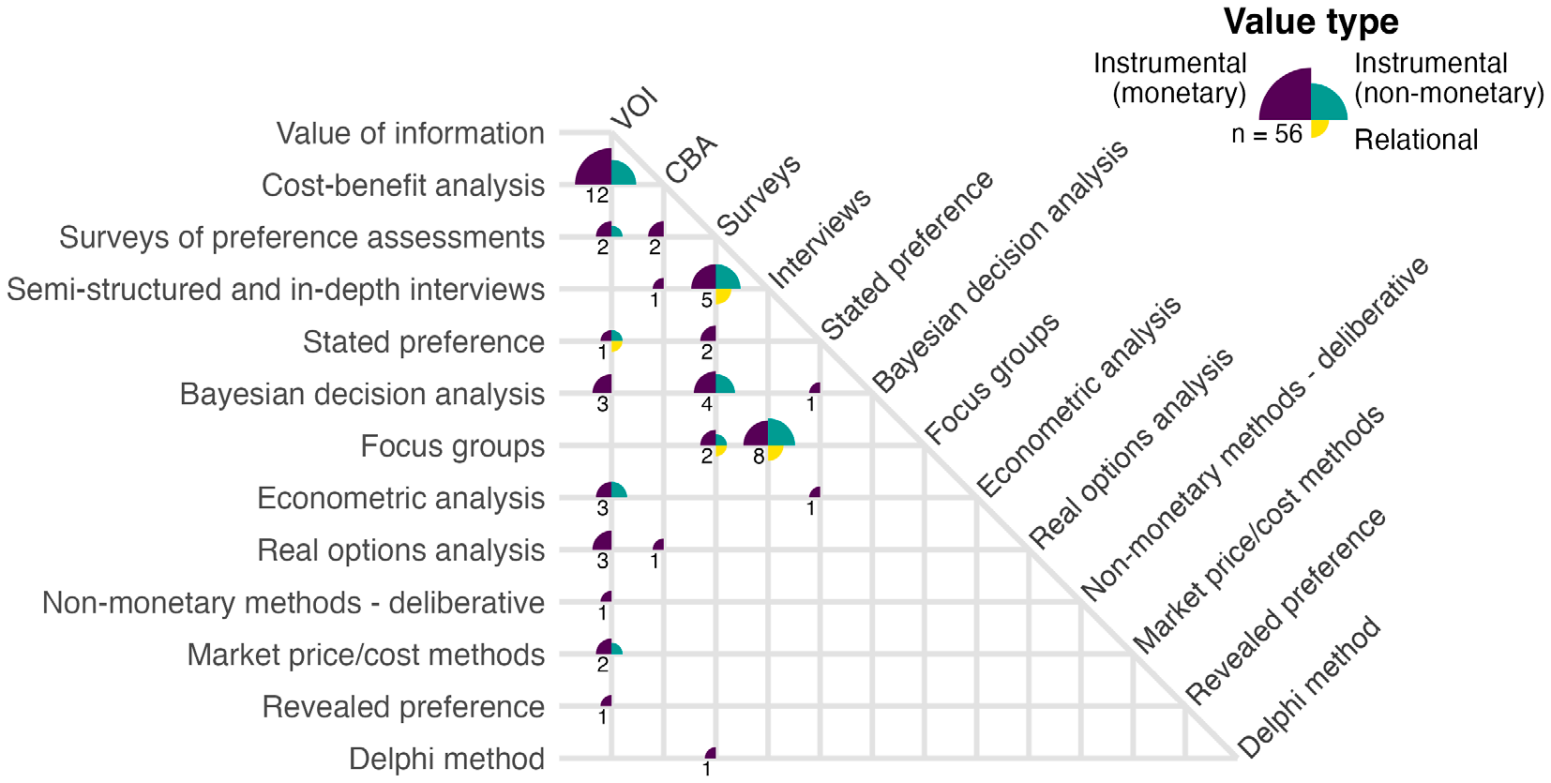
Number of papers applying multiple approaches to valuing ESI. The quadrants and colors at each intersection indicate which value types were examined; the size of the quadrants indicate the proportion of papers that examined each value type. The number in the bottom left quadrant indicates the total number of papers that implemented the combination of methods. The majority of papers (115 out of 171) applied only a single method.

Most studies in our corpus (n = 115) applied only a single method for valuing ESI. Studies that we identified as implementing multiple methods (n = 56) most commonly combined two decision analysis-based methods, particularly VOI with CBA (n = 12 of the 56 studies) (Fig. 3). For example, Fritz et al. (3) applied a VOI framework to estimate benefit, then modeled marginal cost based on CBA to construct their benefit chain model for valuing ESI from hypothetical satellite remote sensing data. Another common pairing combined preference elicitation methods of individual interviews and focus groups (n = 8). For example, Roberts et al. (26) used focus groups/workshops to qualitatively predict the value of weather forecast information for avoiding storm-related drownings in Lake Victoria, then after implementation of a severe weather warning system, followed with user interviews to quantify the realized benefits in lives saved. Of the remaining 36 multiple-method studies, 15 combined VOI with some other method (beyond CBA), 16 combined surveys with some other method (beyond VOI), and 5 applied other combinations. Paired decision analysis-based methods were more common (n = 22) than paired preference elicitation-based (n = 18), but 16 studies combined preference elicitation methods (mostly surveys) with decision analysis methods.

### Societal Benefit Context Areas.

**Fig. 4.**
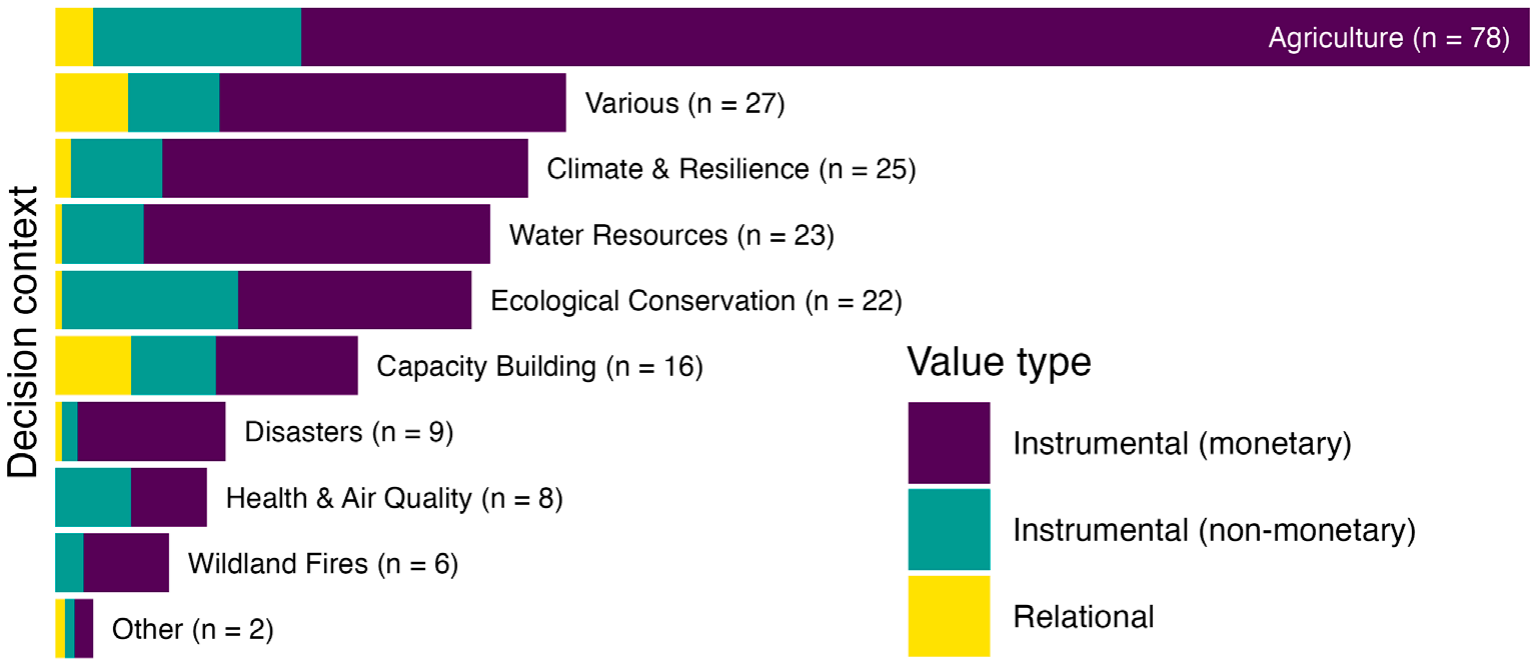
Number of studies investigating value of information in general decision context areas. Some studies examined more than one decision context, thus the total number of specific contexts (216) exceeds the total number of included studies (171). The label “various” indicates studies where decision contexts were broad or undifferentiated; “other” indicates studies where the societal benefit did not fit into any of these contexts. Color indicates value types assessed: instrumental, i.e., means to an end, including both monetary and nonmonetary outcomes, and relational, i.e., deriving from meaningful and often reciprocal relationships among people, nature, and society (*SI Appendix*, Table S1).

To understand how ESI valuation has been applied across sectors and decision contexts within our identified literature, we categorized the primary (and where applicable, secondary) societal benefits into eight general decision context areas, based on existing classes from GEOSS Societal Benefit Areas and NASA’s Applied Sciences Program (Fig. 4). Studies largely focused on the societal benefits of ESI within agriculture contexts, including fisheries and forestry (n = 78, 46% of 171 included studies) (Fig. 4). A smaller but still substantial number of studies examined benefits in context of climate hazards and impacts (n = 25, 15%), water resources (n = 23, 13%), ecological conservation (n = 22, 13%), and capacity building (n = 16, 9%). Societal benefits were least frequently examined in contexts of disaster response (n = 9, 5%), health and air quality (n = 8, 5%), and wildfires (n = 6, 4%). Some studies did not focus deeply on any particular context but rather broadly across various or undifferentiated contexts (“various,” n = 27, 16%), for example, the value of an ocean observing network across many potential ocean uses (27) or the value of Landsat data that did not differentiate among user contexts (28). Two studies focused on ESI benefits in other areas (“other”): one for monitoring pavement infrastructure (29), and one for assessing preferences for living and recreating in disturbed landscapes (30). Across the 171 included studies we observed societal benefits in 216 specific decision contexts (i.e., 45 studies examined societal benefits in more than one context).

**Fig. 5.**
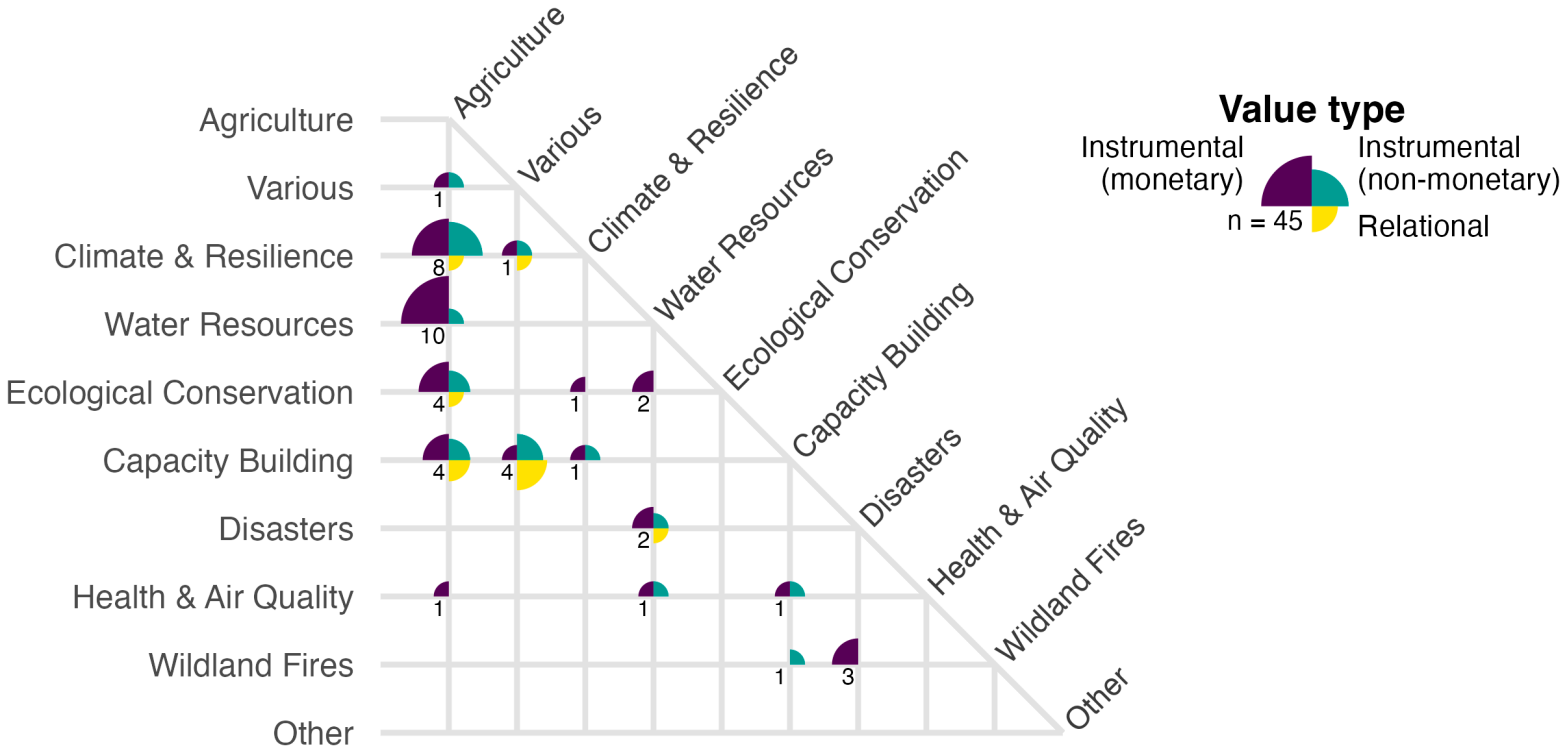
Number of papers valuing ESI in multiple contexts. The quadrants and colors at each intersection indicate which value types were examined; the size of the quadrants indicate how many papers examined that value type. The number in the bottom left quadrant indicates the total number of papers involving that pair of contexts.

Studies that valued ESI across multiple decision contexts (n = 45) most frequently examined agricultural impacts alongside water resources (n = 10), climate (n = 8), ecological conservation (n = 4), and capacity building (n = 4) (Fig. 5). Four studies examined capacity building across various contexts, involving training and supporting groups of stakeholders with diverse roles within their communities, e.g., participatory mapping projects in Nepal (31) and Tanzania (32).

## Discussion

Despite a broad, inclusive search for research on diverse methods for valuing Earth observation information, we found very few examples of evaluations of the societal benefits of ESI. Such a low inclusion rate (1.2%) may reflect a lack of general research on ESI and values, even though our search string was intentionally designed to be inclusive to maximize opportunities to find edge cases in the literature. The paucity of research directly addressing the value of ESI suggests a strong need to better understand how such information is being used to generate societal value, and to identify methods that can effectively assess this value. A key goal of the present study is to promote a more systematic practice of valuation in ESI-based research by highlighting the diversity of methods available to researchers and practitioners.

Our systematic map shows that VOI methods have been the dominant approach to evaluating societal benefits derived from using ESI for decision support (Fig. 2). VOI is a well-established and intuitive method, and Macaulay (20) described a framework for applying VOI to ESI contexts that continues to influence recent research initiatives (e.g., 33). VOI methods are very well suited to situations where a reduction in uncertainty, based on an improved information set, can be expected to drive a clear and measurable improvement in decision outcomes. This is especially the case where costs of a mistake are high, where benefits can be expressed as objective, typically instrumental, quantities, and where the outcome is highly responsive to the set of actions that can be taken. For these reasons, VOI is particularly suited to agricultural contexts (*SI Appendix*, Table S4), where an improved seasonal forecast can inform farmers’ decisions about crop choices and crop management to maximize yield and profit in the face of uncertainty. Conversely, VOI may be a poor fit for cases where the costs of a mistake are externalized, outcomes are difficult to quantify, or values are nonsubstitutable. The fact that the method is intuitive, widely accepted, and can be applied at scale may bias the types of questions we even ask about the value of ESI. Much like someone looking for their lost car keys under a street lamp rather than in the dark alley where they lost them — because that’s where the light is — we risk focusing on questions that are easy to answer with the tools we have, rather than developing methods to fit the questions that are most important to answer.

Further, all valuation methods based on decision analysis (*SI Appendix*, Table S2) necessarily focus on decision outcomes that can be quantified and assume well-identified alternative choices, therefore they also warrant caution. Many decision outcomes valued by individuals and society are impossible to objectively quantify and/or can be qualitatively valued across multiple, potentially incommensurable, value types, and face a range of alternatives of process and decisions that are more complex than many decision analyses typically account for. Preference elicitation methods (*SI Appendix*, Table S2) can more readily account for qualitative and subjective benefits related to ESI-based decision outcomes in instrumental and noninstrumental terms, including relational value. For example, Altamirano et al. (30) surveyed people’s preferences for visiting, living, admiring, and thriving across gradients of landscape disturbance, comparing perceptions of value based on eye-level photos to perceptions based on remote sensing photography. Colloredo-Mansfeld et al. (34), using participatory mapping and in-depth interviews, found that farmers given access to UAV photography perceived their land differently than before, improving relational value through a greater sense of scale and interconnectedness. In aggregating individual preferences elicited through surveys, interviews, and choice experiments, such methods require caveats of their own: they can be subjective and highly context dependent, they are challenging to implement at scale, and they require attention to ensure construct validity, i.e., does the instrument actually measure what it is intended to measure.

In addition to eliciting qualitative and subjective outcomes associated with ESI, preference elicitation methods are well suited to provide insights into the decision process itself, revealing relational values of stewardship, responsibility, and care within a community. For example, Eilola et al. (32) used interviews and focus groups to study how participatory mapping using ESI improved practitioners’ perceptions of work quality, professional competence, participation, and spatial understanding. Gonzalez and Kroger (12) used focus groups and interviews to examine how training in and adoption of remote sensing data improved empowerment and agency of Indigenous people in protecting their land from illegal deforestation. Styers (35) surveyed her undergraduate students to gauge how the incorporation of satellite data into her courses improved student engagement, curiosity, collaborative skills, and learning outcomes. In these cases and others, preference elicitation methods provide insights into how ESI can create value independent of outcome by improving saliency and legitimacy of decision making processes (36).

A few studies bridged the divide between decision-analytic methods and preference-elicitation methods (Fig. 3), which may highlight opportunities to leverage the probabilistic logic of the former while incorporating nuances of individual, societal, and cultural preferences through the latter. For example, in several interrelated studies, Bouma et al. (37–40) applied Bayesian decision analysis to quantify societal benefits of ESI for managing water quality, but leveraged surveys of experts to elicit prior beliefs and expectations of accuracy of the ESI to parameterize the Bayesian analysis, providing insights on how perceptions of accuracy, often elided in a pure VOI analysis, may affect decisions and outcomes. Flipping that script, Luseno et al. (41) used a conceptual Bayesian framework to guide the design of surveys and interviews of pastoralists in Ethiopia and Kenya to understand their preferences around ESI-derived climate forecasts, including the pastoralists’ prior beliefs based on traditional forecasting methods, trust in ESI forecast skill, and likelihood of using the ESI-based forecasts. Their results provide insights into how preferences around cultural norms and traditional ways of knowing may overshadow perceptions of potential gains from adopting ESI, or conversely how such knowledge systems may be marginalized by falsely assuming objectivity of ESI—insights that would likely be lost in a purely decision-analytic framework.

None of the studies in our corpus focused on intrinsic values. Intrinsic value may be difficult to represent in human-led valuations, when defined as values “expressed independently of any reference to people as valuers,” as in the IPBES framework (18). Even in fields such as environmental conservation policy, measurable goals of that policy would necessarily be couched in terms of human-defined outcomes and thus achievement of those goals should be categorized as instrumental value, e.g., remote sensing in support of conservation of Pacific blue whales (9). Yet valuation may be motivated with intention and concern for intrinsic values. Consideration of noninstrumental values is a rapidly developing field in ecosystem service valuation (42), including indigenous and nonanthropocentric world views encompassing more-than-human values (43). Methods to assess noninstrumental societal benefits of ESI will almost certainly increase in sophistication and application as decision makers seek to measure progress toward targets outlined in the UN Sustainable Development Goals and Global Biodiversity Framework (44).

The scientific, political, and commercial structures governing ESI, including whether datasets are publicly accessible or proprietary, freely available or commercial, in part determine who is likely to access benefits from their application (45), but also whose values are represented in the data (and whose are not) (46). Clearly, making ESI data freely available enhances the ability to generate societal benefits; for example, citations and downloads surged for Landsat data following the shift from a paid service to a free and open data policy in 2008, ultimately stimulating billions of dollars in scientific and societal benefits (7, 28). Alvarez León and Gleason (46) analyze how varying property rights can reinforce or challenge assumptions of scientific objectivity and ultimately users’ ability to translate ESI into scientific and societal value. For example, recent data from the European Space Agency (ESA)’s SPOT (Satellite Pour l’Observation de la Terre) mission is commercially available, and users can request that the sensor can be directed to capture imagery of particular regions of commercial interest. As a result, historical SPOT data (through 2015), while freely available through various portals, are skewed toward scenes that were valuable to commercial users at the time of capture. In contrast, Landsat data of USGS are freely and openly available, and its fixed sensing path avoids bias based on commercial interests (46), although systematic gaps in archival georegional coverage may exist due to technical failures and inconsistent data sharing among cooperating nations (47). Any application of ESI over large temporal or spatial scales, whether from free or commercial sources, must acknowledge these technological, commercial, and political sources of potential bias hidden within the data.

Even freely and openly available satellite data come with barriers to technical expertise and capacity that pose significant hurdles to use for many practitioners (48), and poor integration with ground-based and local knowledge hampers development of machine learning algorithms to translate remote sensing imagery into actionable information (49–51). ESI might inadvertently lead to risks where decision-frames lack input from potentially affected communities and where there are asymmetric risks of acting (e.g., where actions can negatively affect groups that have little input to the decisions) (52). Capacity building can help local communities and Indigenous peoples access and incorporate ESI to coproduce knowledge across contexts including conservation (e.g., 53), deforestation (e.g., 12), marine resource management (e.g., 54, 55), and resilience to climate change (41, 56). Such collaborations can reduce power asymmetries and increase agency and self-governance of communities as they seek to address challenges facing indigenous landscapes and territories (12, 56). However, they can also create internal power divisions between tech adopters and nonadopters, potentially resulting in shifts in or loss of cultural values (12).

Positive societal benefits aided by use of ESI were the focus of most of the studies we identified, but a trade-off between information and privacy becomes increasingly relevant as advancements in the quality and quantity of remote sensing data accelerate the ability to identify and monitor objects and people on the ground. In general, remote sensing allows the observer to shift information asymmetry between the observer and the observed, in favor of the observer. Brennan and Macauley (57) describe several important use cases that determine whether the shift in information asymmetry is potentially beneficial or detrimental to society, based on whether the observer and the observed are state actors or private actors (corporations, groups, individuals) and whether the relationship between observer and observed is adversarial or cooperative. The ability of state actors to monitor and enforce compliance with conservation policy, emissions targets, and peace treaties certainly produces societal benefits by enabling cooperation (58); monitoring also holds promise for reducing international conflict (59), supporting human rights (60), and responding to genocide (61). To the extent that an open, transparent government whose laws and regulations reflect the will and consent of the governed, these information asymmetries may actually promote societal benefits, e.g., reduced crime or pollution (57). However, lacking these enabling conditions, ESI could be abused to infringe on rights of privacy and association in personal and public spaces and could enable algorithmic profiling, creating a clear tradeoff between the increasing capabilities of remote sensing technology and the privacy rights of the individual (62).

A growing body of scholarship highlights how remotely sensed and algorithmically produced data embed systematic biases that warrant critical scrutiny (63). For example, conservation algorithms trained on incomplete or unevenly distributed biodiversity data can reproduce existing geographic and sociopolitical biases (e.g., legacies of redlining) resulting in decision frameworks that privilege well-studied regions and taxa (64). Together, these studies underscore that ESI is not a neutral or purely technical substrate; rather, it is a sociotechnical system whose data gaps, algorithmic simplifications, and uneven geographies risk reinforcing misinterpretations of ecological dynamics unless situated within a more reflexive and politically aware framework.

While most of the papers excluded from our target corpus either did not apply ESI data (e.g., spurious matches that were missed during our preliminary screening), or applied ESI data to calculate some other outcome (e.g., using land cover classification data to estimate ecosystem service value, but no further examination of the value of the ESI itself), two categories of excluded papers merit further consideration. These two categories of studies offer clear opportunities for those interested in evaluating the societal benefits of ESI.

First, a number of papers used cost-effectiveness analysis, a close relative of cost–benefit analysis, to demonstrate that an ESI dataset could achieve equal or near-equal performance for a decision context but with less cost (e.g., reduced costs of labor or equipment relative to on-the-ground research) (e.g., 65). We did not include these in our final corpus, reasoning that if the information itself is essentially identical between the ESI and non-ESI alternative, any outcome of a given decision would necessarily be identical, and therefore no additional marginal societal benefit would result from use of the ESI. We acknowledge that in resource-constrained settings, government or NGO cost savings in one area can closely translate into improved societal outcomes in another (e.g., lowering taxes on lower income people, or increasing budgets for social safety nets), but these indirect benefits were not explicitly examined in any of the papers we screened. While these excluded studies focused on a one-time analysis, reduced costs of labor and/or equipment imply the potential for increased frequency of measurement, which would prove valuable for certain types of decision contexts that involve rapidly changing phenomena, e.g., disaster response or wildfire management. We included several studies that explicitly valued the benefits of higher spatial resolution, though we encountered only one study (66) that explicitly accounted for the value of higher temporal resolution. This suggests an opportunity for future valuation studies, especially in light of trends toward increasingly fine temporal resolution of accessible satellite data.

Second, a larger subset of excluded papers compared the ability of ESI to accurately predict on-the-ground phenomena measured by some other means. For example, Marino (67) examined the potential for Sentinel-2 time series imagery to delineate subfields of sunflower crops and found that the image-based vegetation index provided a good proxy for ground-measured crop status; however, the implications for harvest decisions and resulting societal benefit were not explored. Similarly, Andrada et al. (68) demonstrated the efficacy of a drone-based lidar system for rapidly and accurately mapping potential wildfire fuel for forest management, but the authors did not quantify the societal benefit from this valuable scientific information. Such validation studies typically report accuracy scores, e.g., RMSE or AUC, and generally aim to demonstrate the adequacy or superiority of a particular ESI dataset or algorithm over the alternative approach. Importantly, while these studies presented results in terms of scientific value, they did not examine how the improved scientific knowledge would affect the decisions that generate societal benefits — though most included conceptual descriptions of potential decisions or societal value in their conclusions. With often minor additional information or simple economic modeling, many calibration/validation studies could readily translate the improved scientific accuracy of an ESI dataset into a hypothetical or realized decision that results in calculable societal benefits.

Our wide-ranging search string resulted in a large corpus of studies identified as potential candidates for inclusion, but recent developments in machine learning (ML) have made such large screening processes much more feasible. We implemented two distinct ML algorithms in our screening process. First, for all title/abstract screening, we used the Colandr ML-assisted web-based screening tool (24) which uses machine learning and natural language processing to continually predict and sort citations in order of predicted relevance based on user screening decisions. Importantly, Colandr does not decide the disposition of a document — the user is intentionally involved throughout and ultimately makes the decision (24). Second, we generated a training set based on a subset of the full corpus and used this to train an ML algorithm to predict the inclusion/exclusion status of the remaining corpus, identifying nearly 80% of the corpus as likely “excludes.” Because this ML process is recommending the disposition of documents, a low false negative rate (low chance of excluding a relevant document) is critical, though false positives are less problematic, as they are subject to additional human screening. Because our systematic map focused not on study questions or results (typically foregrounded in the title and abstract) but rather on methods (which are often described only vaguely if at all in the abstract), it was difficult to tune the ML model to reduce the false positive rate; however, for studies focused on top-line results typically described in the abstract, such ML methods would likely be far more discerning. Increasingly sophisticated ML algorithms and AI tools such as Elicit, OpenAI Deep Research, and SciSpace Deep Review will almost certainly accelerate rapid systematic evidence synthesis, though the threat of a flood of AI-generated fraudulent literature may drive an arms race in how such reviews are conducted (69).

While our literature search was broad, we restricted it to two databases of academic peer-reviewed literature (Scopus and Web of Science) and the Societal Benefits Library, and did not systematically search gray literature sources. Of the 770 documents identified as candidates for full-text screening, 20 were not retrievable, and 20 more were excluded as not in English; this subset is a tiny fraction of the retrieved papers (2.7%) so omitting these studies is unlikely to substantially affect our results, though a comprehensive search of literature in other languages may provide unique cultural perspectives that are missed in an English-only search. We note that many ESI applications may rely on highly derived, modeled, or processed data, such that remote-sensing terms (e.g., “satellite”) or the name of the initial sensor (e.g., “Landsat”) do not appear in the title, abstract, or keywords, which may limit the citations in our corpus; however, generalizing the search by excluding the ESI terms from the search string would have made the search impossibly large.

## Conclusion

As technological advances increase the cost-effectiveness and capacity for acquisition, storage, and processing of satellite imagery and remote sensing data, ESI will further proliferate in decision support contexts. For example, Canada’s WildfireSat constellation of mission-specific microsatellites, slated to launch in 2029, will image the entirety of Canada in near real time to inform wildfire management, potentially saving billions of dollars in avoided damages as wildfire regimes become increasingly extreme (70). Examining the societal benefits of Earth observation is important to justify existing and future investment (20), promote diffusion of use and applications (71), and identify gaps and priorities for future applications and missions (72, 73).

Methods exist to evaluate ESI contributions across societal benefit areas and value types. However, even as the use of ESI data has grown to encompass a wide range of applications across the globe (71), published peer reviewed studies that attempt to qualitatively or quantitatively assess these contributions remain rare. In the literature we identified that assessed the societal benefits of ESI, the dominance of certain methods, particularly VOI, may have biased inquiry toward quantifiable outcomes and limited the societal contexts in which we have studied them. Instrumental benefits are critical for so many aspects of society, but failing to consider noninstrumental benefits risks underestimating the value that ESI contributes to a thriving society. Looking forward, VOI will continue to be an important tool for ESI valuation, but we have highlighted examples of a broad suite of complementary methods that can potentially explore other questions of value with greater nuance and context relevance.

Our systematic map of the literature revealed a large subset of research that demonstrated the scientific value of particular ESI datasets and models but did not proceed to translate this scientific value into explicit societal value. A major impediment to the uptake of valuation methods as applied to ESI may lie in the gap between science and policy or science and practice. This gap may be attributable in part to lack of in-house social science and policy knowledge to apply valuation methods, and in part to poor engagement between academics and user communities (74). But for the most part, the valuation methods we observe in the literature are well understood by social scientists and practitioners in academic and nonacademic institutions alike. Initiatives and incentives to encourage interdisciplinary collaboration across natural science, social science, and policy or practitioner domains could close the science-policy gap, providing an opportunity to improve our understanding of how ESI translates into societal benefits.

As technical capabilities of ESI instruments and machine learning models rapidly increase, opportunities to translate raw observations into actionable intelligence will multiply. Progress in measuring the instrumental, social, and relational value of ESI is essential to informing this work so that societies can mitigate risks and derive the greatest possible benefit. Here, we have identified concrete examples of qualitative and quantitative valuation methods to measure societal benefits of ESI across a range of decision contexts and value types. By doing so, we hope to inspire other ESI researchers to explore the societal benefit of their own work and contribute to a greater network of valuation practitioners.

## Methods

Our analysis of the literature consisted of five major phases (described in detail below): 1) developing a search string; 2) applying the search string to academic databases to acquire a set of citations; 3) screening citations by the title and abstract; 4) screening the full text of papers that passed the title and abstract screening stage using natural language processing and language models; and 5) coding the papers to identify ESI data source, valuation method, societal benefit area, and value type.

To develop a search string (*SI Appendix*, *Methods*), we focused on three key domains: 1) application of ESI, 2) a decision context or analysis framework in which the ESI is applied, and 3) an expected or observed change in societal benefits based on decision outcome due to use of ESI. The research team collected (via Google Scholar searches) and solicited (via professional networks) a preliminary set of 72 candidate documents, which were screened based on these three domains. Of these 72 candidate documents, 14 were identified as a benchmark set that the research team felt exemplified valuation of ESI. From this benchmark set, we developed a preliminary search string combining the three domains: ESI (e.g., “remote sensing,” “satellite,” “Sentinel,” “Landsat”), decision context (e.g., “management,” “policy,” “cost–benefit,” “contingent valuation”), and societal benefit (e.g., “value,” “benefit,” or “utility” combined with terms such as “societal,” “cultural,” “environmental,” “ecosystem service,” or terms related to GEOSS societal benefit areas). The preliminary set of terms was used to collect citations (title, abstract, authors, metadata) from Web of Science (n = 1,158). We applied the functionality of the litsearchr package in R (75) to this preliminary citation set, using text mining and keyword co-occurrence networks to identify additional terms to increase the inclusion of our search string. The final search string (*SI Appendix*, Fig. S1 and *Methods*) was used to collect citations from Web of Science (January 26, 2024, n = 9,488) and Scopus (February 4, 2024, n = 18,585), including all 14 benchmark papers. In addition to these two citation sets we included a curated set of citations from the USGS Joint Societal Benefits of Earth Observation Digital Library (https://doi.sciencebase.gov/hd/#/geo-value?p=0&l=50&yearMin=1863&yearMax=2024) (SBL, n = 258). See Fig. 1 for PRISMA flow diagram.

The results of the search (Web of Science and Scopus) were then cleaned. Citations noted as conference abstracts or proceedings (n = 1,030 and n = 4,109 respectively) were dropped. Then, citations with missing title, author, abstract, or digital object identifier (DOI) field (n = 319 and n = 1,226 respectively) were dropped. After resolving minor differences among titles, author names, and DOI fields, 6,840 duplicate citations were removed from the combined citation set. The resulting set of 14,807 distinct citations were subjected to a preliminary screening to remove identified spurious matches (n = 984), leaving n = 13,823 citations for screening and analysis (Fig. 1).

Screening was performed in two stages, the first to label a training set to train a supervised learning classification model, and the second to apply the classification model to predict relevant papers within the larger corpus (Fig. 2). In the first stage, the citations from the SBL and a random sample of ~1000 citations from the Web of Science/Scopus corpus were subjected to title/abstract screening, and then full-text screening on the title/abstract “include” papers, based on a set of inclusion criteria (See *SI Appendix*, *Methods* for screening criteria). All title/abstract screening was performed using the Colandr web-based screening application (24), which uses machine learning and natural language processing to continually predict and sort citations in order of predicted relevance based on user screening decisions. As a user screens documents and codes them as “include” or “exclude,” Colandr develops a predictive model and iteratively sorts the remaining unscreened documents, presenting the user with the most likely relevant documents early. As fewer and fewer relevant documents are identified, and the inclusion rate approaches zero, the user can opt to establish an early-stopping rule as the remainder of the corpus is deemed increasingly unlikely to be relevant. For this initial stage, we did not set an early-stopping rule, and simply screened all citations.

The resulting set from the first stage was then used to train a classification model based on the XLNet generalized autoregressive pretraining algorithm, which considers all permutations of dependencies between sets of words in the citation titles and abstracts to “understand” the context (76), to classify citations in the remainder of the corpus as either “include” or “exclude”. The predicted “include” citations were then title/abstract screened (using Colandr) and those that passed were then screened based on the full text. The include/exclude classification model showed a low false negative rate (1.2%, sensitivity 92.3%) on the training data, but to ensure this held true of the larger document set, a random sample of 1000 predicted “excludes” was uploaded to Colandr. After screening 200 of these documents and finding no relevant matches to our screening criteria despite Colandr’s ability to prioritize relevant articles, this screening phase was stopped early. While the classifier’s false positive rate was higher (27.1%, specificity 71.2%), these false positives were subject to title/abstract screening so were not a concern. Of the 13,823 unique citations retrieved from Scopus, Web of Science, and the SBL, our screening process resulted in only n = 170 documents that met all screening criteria for inclusion (*SI Appendix*, *Methods*) in our corpus, for an inclusion rate of 1.2%. One additional reference was added postscreening, at the recommendation of one of the coauthors, for a final corpus of n = 171 documents.

Documents included in the final corpus were manually coded based on reading the full text to identify valuation methods and value types according to *SI Appendix*, Tables S1 and S2. All analysis and figures were generated using R statistical software version 4.4.1 (77) and the tidyverse metapackage version 2.0.0 (78).

## Supplementary Material

Appendix 01 (PDF)

## Data Availability

Data retrieved for review, data coded for analysis, code for processing and analyzing data have been deposited in Knowledge Network for Biocomplexity (79) and GitHub (80).
