# Supplementary material for: A systematic map of methods for assessing societal benefits of Earth science information
Source: Proc Natl Acad Sci U S A. 2026 Feb 6;123(6):e2524370123. doi: 10.1073/pnas.2524370123 (PMC12890935; doi:10.1073/pnas.2524370123)
Supplement: Supplementary file 1 — Appendix 01 (PDF) [file pnas.2524370123.sapp.pdf]

PNAS

Supporting Information for:
A Systematic Map of Methods for Assessing Societal Benefits of Earth Science Information

Casey C. O'Hara, Mabel Baez-Schon, Rebecca Chaplin-Kramer, Samantha H. Cheng, Alejandra Echeverri, Gillian L. Galford, Rachelle K. Gould, Cristina L. Mancilla, Maura C. Muldoon, Gerald G. Singh, Priscilla Baltezar, Yusuke Kuwayama, Stephen Polasky, Amanda D. Rodewald, Richard P. Sharp, Elizabeth J. Tennant, Jiaying Zhao, Benjamin S. Halpern

List of Supporting Materials

Tables S1 - S4

Figure S1

Supporting Methods

Supporting References

Value domains

Table S1. Definitions of value domains. This table is based on the work of Himes et al. (1) on valuation of nature, and adapted to account for potential value derived from Earth science information. In nearly all cases, the value of ESI is based on the degree to which the expected outcome of a decision is improved by incorporating ESI into the decision. Where applicable, we have broadened ecosystems, biodiversity, and ecosystem services to include social and natural features and outcomes that are improved by incorporation of ESI into decision making processes.

Value Domain	Core Meaning	Salient Articulation	Examples in included corpus (see Table S3)
Instrumental	Values of entities or processes important as means to achieve human ends or satisfy human preferences (in principle replaceable, albeit not always in practice)	Means to an end (mostly intended as usefulness for humans, utility, or benefits, sometimes also for other-than-human beings); Leading to satisfaction of needs, preferences, interests, and desires; Nature's value as a resource, for ecosystem services, as an asset, capital, or property	Reduced polio transmission and reduced health cost due to improved targeting of populations (Borowitz et al. 2023); increased consumer surplus of crops due to improved weather forecasts (Cooke and Golub 2020); avoided losses from improved wildfire suppression (Herr et al. 2020)
Intrinsic	Values of entities expressed independently of any reference to people as valuers (including values associated with entities worth protecting as ends in and of themselves)	Defined negatively as noninstrumental value; Value of something that is an end in itself, has agency; Objective value or value independent of being valued or recognized by (human) valuer—inherent properties of something; Regardless of importance or usefulness to humans; Inherent moral value of natural beings (right to exist)	Not observed in literature
Relational	Values of meaningful and often reciprocal human relationships—beyond means to an end—with nature (often specified as a particular landscape, place, species, forest, etc.) or society, and among people through nature or society	Values of or deriving from desirable, meaningful, just and reciprocal relationships with "nature" or between people through nature; Values relative to or deriving from relationships that are constituent parts of identity (cultural, individual or collective); Values relative to or deriving from relationships that are constituent elements for living a "good life"; Values associated with sense of place, including interconnection of cultural and sacred landscapes; Values associated with care for or about specific landscapes, places, human and other-than-humans; Value of nature as a point of connection among people, binding communities together and supporting social networks, such as in traditional markets	Increased agency of Indigenous communities for monitoring and enforcing illegal deforestation (Gonzalez et al. 2023); sense of community and quality of life through common understanding of decision contexts (Sawyer et al. 2022)

Valuation methods

Table S2. Valuation methods with potential application to valuing Earth science information. Preference elicitation methods are defined in Arias-Arevalo et al. (2) and adapted to apply to Earth science information.

Category	Method	Description	Examples (see Table S3)
Decision analysis (quantitative)	Bayesian Decision Analysis	Information is used to update a decision-maker's prior beliefs about potential outcomes, generally to reduce uncertainty and/or variance in expected outcome. Accounts for decision-maker's prior beliefs about the quality of information.	Brathwaite and Saleh 2013, Bouma et al. 2011, Luseno et al. 2003
	Value of Information	Subset of Bayesian Decision Analysis. Compares expected/realized value of outcome with ESI vs counterfactual. Decision-maker's prior beliefs not addressed.	Forney et al. 2012, Herr et al. 2020, Macauley 2006, Oddo and Bolten 2019,
	Cost-benefit analysis	Compares expected/realized value of outcome with new information to the cost of obtaining that information. Flows of benefits and costs over time are expressed on a common basis in terms of their net present value. Benefits can be *avoided costs* e.g., use of ESI helps avoid loss of crop profits	Li et al. 2017, Morretta et al. 2023, Vuolo et al. 2015
	Real options analysis	Real options value based on the right, but not obligation, to act in the future based on resolution of uncertain outcomes.	Cooke and Golub 2020, Fuss et al. 2006
	Econometric analysis	Information is explicitly included in econometric analysis as an independent/predictor variable; its effect on outcome variable (monetary or other benefit) is used to determine value of information	Bridges et al. 2018, Diana and Farida 2021
Preference elicitation: Monetary valuation methods (quantitative)	Market price-based methods	Uses prices of ESI traded in markets (e.g., commercial satellite imagery) as a proxy for its monetary value	Harris et al. 2000, Hautala et al. 2008
	Market cost-based methods	Estimate the costs that are averted due to the ESI application. The production function estimates the degree to which ESI contributes to the delivery of a marketed good	Stroming et al. 2020
	Stated preference (contingent valuation; choice modeling)	Constructs hypothetical markets and asks about willingness to pay (WTP) to obtain a specified ESI, or willingness to accept (WTA) giving it up. Choice modelling infers WTP through trade-offs incurred when choosing between alternatives with different levels of ESI and costs	Jabbour et al. 2020, Kim et al. 2022
	Revealed preference (travel cost; hedonic pricing)	Travel cost method analyses individual choices in markets related to ESI. Travel cost methods use the costs of travel to a natural area as a measure of the value of recreation. Hedonic pricing method reveals the monetary value of ESI mainly through house prices	Newbold et al. 2022
	Benefit transfer	Estimates the monetary value of ESI by transferring a measure estimated in a similar context	none found
Preference elicitation: Monetary valuation methods - Mixed (quantitative and qualitative)	Economic field experiments	Experiments developed in naturally-occurring settings aimed at analysing behaviour and decision making (e.g. choices influenced by reciprocity, norms, altruism and uncertainty)	none found
	Deliberative economic valuation	Combines stated preference valuation methods with elements of deliberative processes	none found
Preference elicitation: Non-monetary valuation methods (quantitative)	Surveys of preference assessments	Surveys aiming to rank or rate preferences for ESI. Used to analyse perceptions, knowledge and values of ESI demand/use	Amegnaglo et al. 2022, Diana and Ibrahim 2020, Safar et al. 2022
	Photo-elicitation surveys	Visual elements (e.g. photographs, pictures) are included in surveys to assess individuals' perception of ESI values and preferences towards landscape views	Altamirano et al. 2020, Colloredo et al. 2020
	Time use surveys	Captures individuals' willingness to give up time (WTT) for activities that promote ESI production/maintenance	none found

Category	Method	Description	Examples (see Table S3)
	Psychometric surveys	Elicits data on individual attitudes, views, reported behaviour, motivations and values towards ESI	none found
Preference elicitation: Non-monetary valuation methods - mixed (qualitative and quantitative)	Delphi Method	Uses expert opinion to reach an agreed conclusion. It may involve quantitative and qualitative assessments	Taramelli et al. 2020
	Q Methodology	Analyses subjectivity (i.e. attitudes, shared perceptions and worldviews) through individual ranking of statements. Common worldviews are elucidated through factor analysis	none found
Preference elicitation: Non-monetary valuation methods (qualitative)	Semi-structured and in-depth interviews	In-depth interviews capture how people value or understand something. In a semi-structured interview, the researcher orients the conversation to specific topics	Boyd et al. 2022, Bruno Soares 2017, Luseno et al. 2003
	Participatory observation	The researcher gets involved with people in their natural environment. Aimed at analysing people's cultural behaviours and interactions	none found
	Participant diaries	Participants are asked to make regular records or narrative descriptions of personal experiences. Aimed at exploring thoughts, feelings and understandings of a topic of interest to the research	none found
	Photo-voice	Stakeholders take their own photographs of different features of ecosystems and landscapes (e.g. ES). Useful to integrate the perceptions of marginalised social groups	none found
	Focus groups	An externally-guided group discussion about a topic. Aimed at discovering different positions and to explore how participants interact in discussion	Eilola et al. 2023, Roberts et al. 2022, Sciavon et al. 2023
Preference elicitation: Non-monetary valuation methods - deliberative	Citizen juries	Groups of representative citizens – randomly chosen - act as jurors to consider issues of public importance	none found
	Deliberative focus groups	Similar to focus groups, but may have more than one reunion, and have an emphasis on consensus and collective decision	none found
	Participant action research	People work collaboratively with researchers in knowledge co-production. Aimed at finding solutions to problems of common interest	Seelan et al. 2003, Seielstad et al. 2002
	Participatory rural appraisal; rapid rural appraisal	Promotes local knowledge and enables local people to make their own appraisals, analysis and plans	Parajuli et al. 2020
	Participatory scenario planning	A tool for analysing future prospects of change in ESI and its trade-offs. Involves the participatory identification of storylines, drivers of change, uncertainties and scenario outcomes	none found
	Mediated modelling	Combines dynamic system modelling with stakeholder participation, aimed at creating a shared model of alternative outcomes	none found
	Deliberative mapping	Stakeholders create a map via consensus, indicating valuable ES and landscape futures	none found

PNAS

Included papers

Table S3. Corpus of papers included in analysis.

Reference	ESI source	Valuation method(s)	Decision context	Value type(s)
R. M. Adams, et al., The benefits to Mexican agriculture of an El Niño-southern oscillation (ENSO) early warning system. Agricultural and Forest Meteorology 115, 183–194 (2003).	ENSO early warning system (hypothetical)	Value of information; Bayesian decision analysis	Agriculture	Instrumental (monetary)
R. M. Adams, et al., Value of Improved Long-Range Weather Information. Contemporary Economic Policy 13, 10–19 (1995).	ENSO early warning system (hypothetical)	Value of information; Bayesian decision analysis	Agriculture	Instrumental (monetary)
A. Altamirano, et al., Landscape disturbance gradients: The importance of the type of scene when evaluating landscape preferences and perceptions. Land (2020).	aerial images	Surveys of preference assessments	Other	Instrumental (non-monetary); Relational
C. J. Amegnaglo, K. A. Anaman, A. Mensah-Bonsu, E. E. Onumah, F. Amoussouga Gero, Contingent valuation study of the benefits of seasonal climate forecasts for maize farmers in the Republic of Benin, West Africa. Climate Services 6, 1–11 (2017).	Seasonal forecasts (hypothetical)	Stated preference; Surveys of preference assessments	Agriculture	Instrumental (monetary)
R. A. Asiyanbola, An evaluation of public servant awareness and use of gis/remote sensing in africa-nigeria. South African Journal Of Geomatics (2018).	remote sensing (hypothetical)	Surveys of preference assessments	Capacity Building	Instrumental (monetary)
H. Awada, et al., Assessing the performance of a large-scale irrigation system by estimations of actual evapotranspiration obtained by landsat satellite images resampled with cubic convolution. International Journal Of Applied Earth Observation And Geoinformation (2019).	Landsat	Value of information	Agriculture; Water Resources	Instrumental (monetary)
B. A. Babcock, The Value of Weather Information in Market Equilibrium. American J Agri Economics 72, 63–72 (1990).	seasonal forecast (hypothetical)	Value of information; Bayesian decision analysis	Agriculture	Instrumental (monetary)
J. Bacenetti, et al., May smart technologies reduce the environmental impact of nitrogen fertilization? A case study for paddy rice. Science Of The Total Environment (2020).	Sentinel	Value of information	Agriculture; Climate & Resilience	Instrumental (monetary); Instrumental (non-monetary)
J. F. Bard, A. Watkins, Improved rangeland management with an earth resource survey system. Technological Forecasting And Social Change (1983).	Earth Resource Survey system	Value of information; Cost-benefit analysis	Agriculture	Instrumental (monetary); Instrumental (non-monetary)
J. Berenter, I. Morrison, J. M. Mueller, Valuing User Preferences for Geospatial Fire Monitoring in Guatemala. Sustainability 13, 12077 (2021).	SIGMA-I	Stated preference	Wildland Fires	Instrumental (monetary)
E. Bergseng, H. O. Ørka, E. Næsset, T. Gobakken, Assessing forest inventory information obtained from different inventory approaches and remote sensing data sources. Annals Of Forest Science (2015).	airborne laser scanning	Value of information	Agriculture	Instrumental (monetary)
R. Bernknopf, Agricultural case studies for measuring the value of information of earth observation and other geospatial information for decisions. Geovalue: The Socioeconomic Value Of Geospatial Information (2017).	Landsat;MODIS;AWiFS;GRACE	Value of information; Econometric analysis	Agriculture; Water Resources	Instrumental (monetary)
R. L. Bernknopf, W. M. Forney, R. P. Raunikar, S. K. Mishra, Estimating the benefits of land imagery in environmental applications: a case study in nonpoint source pollution of groundwater. The Value Of Information: Methodological Frontiers And New Applications In Environment And Health (2012).	MRLI (Landsat)	Value of information	Water Resources; Agriculture	Instrumental (monetary)

Reference	ESI source	Valuation method(s)	Decision context	Value type(s)
R. Bernknopf, et al., The Value of Remotely Sensed Information: The Case of a GRACE-Enhanced Drought Severity Index. Weather, Climate, and Society 10, 187–203 (2018).	GRACE	Bayesian decision analysis	Climate & Resilience	Instrumental (monetary)
R. Bernknopf, D. S. Brookshire, P. T. Ganderton, "The Role Of Geoscience Information In Reducing Catastrophic Loss Using A Web-Based Economics Experiment" (2003).	Simulated	Stated preference	Disasters	Instrumental (monetary)
R. L. Bernknopf, D. S. Brookshire, M. McKee, D. R. Soller, Estimating the Social Value of Geologic Map Information: A Regulatory Application. Journal of Environmental Economics and Management 32, 204–218 (1997).	geologic map	Bayesian decision analysis	Various	Instrumental (monetary)
R. Bernknopf, C. Shapiro, Economic Assessment of the Use Value of Geospatial Information. IJGI 4, 1142–1165 (2015).	MRLI (Landsat)	Value of information	Agriculture; Water Resources	Instrumental (monetary)
R. Bernknopf, A. Steinkruger, Y. Kuwayama, "Earth Observations Can Enable Cost-Effective Conservation of Eastern North Pacific Blue Whales: A Value of Information Analysis" (Resources for the Future, 2021).	Remotely sensed data and information	Value of information	Ecological Conservation	Instrumental (monetary); Instrumental (non-monetary)
P. Bettinger, et al., Stakeholder perceptions on the need for updated tree species distribution maps. Forests (2021).	remote sensing	Surveys of preference assessments	Agriculture	Instrumental (monetary)
I. Bobojonov, A. Aw-Hassan, R. Sommer, Index-based insurance for climate risk management and rural development in syria. Climate And Development (2014).	MODIS	Econometric analysis	Agriculture; Climate & Resilience	Instrumental (monetary)
M. Borowitz, J. Zhou, K. Azelton, I.-Y. Nassar, Examining the value of satellite data in halting transmission of polio in Nigeria: A socioeconomic analysis. Data & Policy 5, e16 (2023).	DigitalGlobe	Value of information	Health & Air Quality; Capacity Building	Instrumental (monetary); Instrumental (non-monetary)
S.-A. Boukabara, R. N. Hoffman, Optimizing observing systems using aspen: An analysis tool to assess the benefit and cost effectiveness of observations to earth system applications. Bulletin Of The American Meteorological Society (2022).	various	Cost-benefit analysis	Various	Instrumental (monetary)
J. A. Bouma, O. J. Kuik, H. J. van der Woerd, A. G. Dekker, The value of Earth Observation for marine water quality management in Remote Sensing of Environment, (2009), pp. 1–4.	EO data	Bayesian decision analysis; Surveys of preference assessments	Agriculture; Ecological Conservation	Instrumental (monetary)
J. A. Bouma, H. J. van der Woerd, O. J. Kuik, Assessing the value of information for water quality management in the North Sea. Journal of Environmental Management 90, 1280–1288 (2009).	Global Earth Observation (hypothetical)	Bayesian decision analysis; Surveys of preference assessments	Ecological Conservation; Agriculture	Instrumental (monetary); Instrumental (non-monetary)
J. A. Bouma, O. Kuik, A. G. Dekker, Assessing the value of Earth Observation for managing coral reefs: An example from the Great Barrier Reef. Science of The Total Environment 409, 4497–4503 (2011).	Ocean color satellite data (hypothetical)	Bayesian decision analysis; Surveys of preference assessments	Ecological Conservation	Instrumental (monetary); Instrumental (non-monetary)
J. Bouma, O. Kuik, A. Dekker, The Value of Earth Observation for Managing the Great Barrier Reef. (2009).	Ocean color satellite data (hypothetical)	Bayesian decision analysis; Surveys of preference assessments	Ecological Conservation	Instrumental (monetary); Instrumental (non-monetary)
A. Bounfour, E. Lambin, How valuable is remotely sensed information? The case of tropical deforestation modelling. Space Policy (1999).	Landsat	Cost-benefit analysis	Ecological Conservation	Instrumental (monetary)
D. S. Boyd, et al., Citizen science for earth observation (citzens4eo): Understanding current use in the uk. International Journal Of Remote Sensing (2022).	Maxar WorldView imagery	Semi-structured and in-depth interviews; Surveys of preference assessments	Various; Capacity Building	Instrumental (monetary); Instrumental (non-monetary); Relational
J. Brathwaite, J. H. Saleh, Bayesian framework for assessing the value of scientific space systems: Value of information approach with application to earth science spacecraft. Acta Astronautica 84, 24–35 (2013).	Hypothetical hurricane forecast	Bayesian decision analysis	Climate & Resilience	Instrumental (monetary)

PNAS

Reference	ESI source	Valuation method(s)	Decision context	Value type(s)
D. J. Bridges, et al., Accuracy and impact of spatial aids based upon satellite enumeration to improve indoor residual spraying spatial coverage. Malaria Journal (2018).	Satellite imagery	Econometric analysis	Health & Air Quality	Instrumental (non-monetary)
M. Bruno Soares, Assessing the usability and potential value of seasonal climate forecasts in land management decisions in the southwest UK: challenges and reflections. Adv. Sci. Res. 14, 175–180 (2017).	seasonal climate forecast	Focus groups; Semi-structured and in-depth interviews	Agriculture	Instrumental (monetary)
A. Burgin, Compliance with european union environmental law: An analysis of digitalization effects on institutional capacities. Environmental Policy And Governance (2020).	Copernicus;satellite;digitalization	Semi-structured and in-depth interviews	Capacity Building	Instrumental (monetary)
V. E. Cabrera, D. Letson, G. Podestá, The value of climate information when farm programs matter. Agricultural Systems 93, 25–42 (2007).	ENSO forecasts	Value of information	Agriculture	Instrumental (monetary)
A. Chamuah, R. Singh, Securing sustainability in indian agriculture through civilian uav: a responsible innovation perspective. Sn Applied Sciences (2020).	UAV	Semi-structured and in-depth interviews	Agriculture	Instrumental (monetary); Instrumental (non-monetary); Relational
C.-C. Chen, B. McCarl, H. Hill, Agricultural Value of ENSO Information under Alternative Phase Definition. Climatic Change 54, 305–325 (2002).	ENSO forecasts	Value of information	Agriculture; Climate & Resilience	Instrumental (monetary)
B. R. Christensen, Use of UAV or remotely piloted aircraft and forward-looking infrared in forest, rural and wildland fire management: evaluation using simple economic analysis. N.Z. j. of For. Sci. 45, 16 (2015).	UAV	Surveys of preference assessments; Cost-benefit analysis	Disasters; Wildland Fires	Instrumental (monetary)
F. Collard, C. Haritchabalet, Valuing satellite systems to support fishing in a dynamic competitive model. Applied Economics (2012).	hypothetical satellite system to detect fish	Value of information	Agriculture	Instrumental (monetary)
M. Colloredo-Mansfeld, F. J. Laso, J. Arce-Nazario, Drone-based participatory mapping: Examining local agricultural knowledge in the galapagos. Drones (2020).	UAV	Semi-structured and in-depth interviews; Surveys of preference assessments	Agriculture; Ecological Conservation	Instrumental (monetary); Instrumental (non-monetary); Relational
R. Cooke, et al., Using the social cost of carbon to value earth observing systems. Climate Policy (2017).	CLARREO	Value of information; Real options analysis	Climate & Resilience	Instrumental (monetary)
R. Cooke, A. Golub, Market-based methods for monetizing uncertainty reduction. Environ Syst Decis 40, 3–13 (2020).	SMAP	Real options analysis; Value of information	Agriculture	Instrumental (monetary)
R. Cooke, B. A. Wielicki, D. F. Young, M. G. Mlynczak, Value of information for climate observing systems. Environ Syst Decis 34, 98–109 (2014).	CLARREO	Value of information	Climate & Resilience	Instrumental (monetary)
C. J. Costello, R. M. Adams, S. Polasky, The Value of El Niño Forecasts in the Management of Salmon: A Stochastic Dynamic Assessment. American J Agri Economics 80, 765–777 (1998).	ENSO forecasts	Value of information	Agriculture; Ecological Conservation	Instrumental (monetary)
L. Cristini, et al., Cost and value of multidisciplinary fixed-point ocean observatories. Marine Policy 71, 138–146 (2016).	FixO3 ocean observatory network	Cost-benefit analysis	Climate & Resilience; Ecological Conservation	Instrumental (monetary)
F. Destandau, A. P. Diop, An analysis of the value of additional information provided by a water quality measurement network. Journal of Water Resource and Protection 8, 767 (2016).	Water quality monitoring networks	Bayesian decision analysis	Water Resources; Ecological Conservation	Instrumental (monetary)
F. Destandau, Y. Zaiter, Spatio-temporal design for a water quality monitoring network maximizing the economic value of information to optimize the detection of accidental pollution. Water Resources and Economics 32, 100156 (2020).	Water quality monitoring networks	Value of information	Water Resources; Ecological Conservation	Instrumental (monetary)
G. Di Lallo, P. Mundhenk, M. Marchetti, M. Köhl, Understanding measurement reporting and verification systems for redd+ as an investment for generating carbon benefits. Forests (2017).	Satellite imagery;lidar	Cost-benefit analysis; Value of information	Agriculture; Climate & Resilience	Instrumental (monetary); Instrumental (non-monetary)

PNAS

Reference	ESI source	Valuation method(s)	Decision context	Value type(s)
I. Diafas, P. Panagos, L. Montanarella, Willingness to Pay for Soil Information Derived by Digital Maps: A Choice Experiment Approach. Vadose Zone Journal 12, 1–8 (2013).	airborne hyper-spectral among other ground-based systems	Stated preference	Agriculture; Water Resources	Instrumental (monetary)
S. R. Diana, F. Farida, Applying bag of words approach to determine remote sensing technology acceptance among smallholder plantations. Arab Gulf Journal Of Scientific Research (2023).	Remote sensing	Focus groups; Semi-structured and in-depth interviews	Agriculture	Instrumental (monetary); Instrumental (non-monetary)
S. R. Diana, F. Farida, Economic Potential of Oil Palm Plantation Using Remote Sensing-Based Technology in Indonesia. ajtm 14, 19–34 (2021).	SPOT	Econometric analysis	Agriculture	Instrumental (monetary)
S. R. Diana, I. M. Ibrahim, Intangible economic benefit of remote sensing data in Indonesia. IJRBS 9, 150–159 (2020).	remote sensing	Surveys of preference assessments; Semi-structured and in-depth interviews	Agriculture	Instrumental (monetary); Instrumental (non-monetary)
E. Diez, B. S. McIntosh, Organisational drivers for, constraints on and impacts of decision and information support tool use in desertification policy and management. Environmental Modelling & Software (2011).		Semi-structured and in-depth interviews	Agriculture	Instrumental (monetary); Instrumental (non-monetary)
H. M. I. Ebaid, S. S. Ismail, Lake nasser evaporation reduction study. Journal Of Advanced Research (2010).	remote sensing and GIS	Value of information	Water Resources	Instrumental (non-monetary)
S. Eilola, N. Kayhko, N. Fagerholm, Lessons learned from participatory land use planning with high-resolution remote sensing images in tanzania: Practitioners' and participants' perspectives. Land Use Policy (2021).	satellite imagery; aerial imagery	Semi-structured and in-depth interviews; Focus groups	Various; Capacity Building	Instrumental (non-monetary); Relational
Y. S. Eom, J. H. Hong, Measuring the economic benefits of an environmental monitoring satellite project: The value of information approach. Space Policy 29, 203–209 (2013).	GEMS	Stated preference	Health & Air Quality	Instrumental (monetary); Instrumental (non-monetary)
J. R. B. Fisher, E. A. Acosta, P. J. Dennedy-Frank, T. Kroeger, T. M. Boucher, Impact of satellite imagery spatial resolution on land use classification accuracy and modeled water quality. Remote Sensing In Ecology And Conservation (2018).	Digital Globe;Landsat	Cost-benefit analysis	Water Resources	Instrumental (monetary)
P. D. Fisher, M. Abuzar, M. A. Rab, F. Best, S. Chandra, Advances in precision agriculture in south-eastern australia. I. A regression methodology to simulate spatial variation in cereal yields using farmers' historical paddock yields and normalised difference vegetation index. Crop & Pasture Science (2009).	Landsat;SPOT	Value of information	Agriculture	Instrumental (monetary)
J. K. Fletcher, et al., Tropical africa's first testbed for high-impact weather forecasting and nowcasting. Bulletin Of The American Meteorological Society (2023).	African Science for Weather Information and Forecasting Techniques	Surveys of preference assessments	Climate & Resilience; Capacity Building	Instrumental (monetary); Instrumental (non-monetary)
J. Florens, C. Foucher, Pollution monitoring: Optimal design of inspection - an economic analysis of the use of satellite information to deter oil pollution. Journal Of Environmental Economics And Management (1999).	Satellite imagery	Cost-benefit analysis	Ecological Conservation	Instrumental (monetary); Instrumental (non-monetary)
W. M. Forney, R. Raunikar, S. Mishra, R. Bernknopf, An economic value of remote-sensing information: Application to agricultural production and maintaining ground waterquality in 2012 Socio-Economic Benefits Workshop: Defining, Measuring, and Communicating the Socio-Economic Benefits of Geospatial Information, (IEEE, 2012), pp. 1–6.	MRLI (Landsat)	Value of information	Water Resources; Agriculture	Instrumental (monetary)
C. Fraccaroli, et al., Climate data for the european forestry sector: From end-user needs to opportunities for climate resilience. Climate Services (2021).	Copernicus Climate Change Services (C3S)	Semi-structured and in-depth interviews	Agriculture; Climate & Resilience	Instrumental (non-monetary)

Reference	ESI source	Valuation method(s)	Decision context	Value type(s)
J. Francis, M. Disney, S. Law, Monitoring canopy quality and improving equitable outcomes of urban tree planting using lidar and machine learning. Urban Forestry & Urban Greening (2023).	lidar	Value of information	Agriculture; Climate & Resilience	Instrumental (non-monetary); Relational
S. Fritz, R. J. Scholes, M. Obersteiner, J. Bouma, B. Reyers, A Conceptual Framework for Assessing the Benefits of a Global Earth Observation System of Systems. IEEE Systems Journal 2, 338–348 (2008).		Value of information; Cost-benefit analysis	Various	Instrumental (monetary); Instrumental (non-monetary)
S. Fuss, J. Szolgayova, M. Obersteiner, A real options approach to satellite mission planning. Space Policy (2008).	Satellite imagery	Real options analysis	Disasters	Instrumental (monetary)
M. Glantz, The value of a Long-Range weather Forecast for the west African sahel. 58 (1977).	hypothetical long-range weather forecast system	Surveys of preference assessments; Value of information	Agriculture	Instrumental (monetary); Instrumental (non-monetary)
N. C. Gonzalez, M. Kroger, The adoption of earth-observation technologies for deforestation monitoring by indigenous people: Evidence from the amazon. Globalizations (2023).	forest monitoring technology (satellite, drone)	Focus groups; Semi-structured and in-depth interviews	Agriculture; Capacity Building	Instrumental (non-monetary); Relational
N. E. Graham, K. P. Georgakakos, C. Vargas, M. Echevers, Simulating the value of El Niño forecasts for the Panama Canal. Advances in Water Resources 29, 1665–1677 (2006).	NINO3 SST ENSO forecast	Value of information	Water Resources	Instrumental (monetary)
A. Haara, A. Kangas, S. Tuominen, Economic losses caused by tree species proportions and site type errors in forest management planning. Silva Fennica (2019).	aerial imagery; satellite imagery; airborne laser scanning	Value of information	Agriculture	Instrumental (monetary)
D. L. Halsing, K. Theissen, R. Bernknopf, A cost-benefit analysis of The National Map. Circular (2004).	National Map	Cost-benefit analysis	Various	Instrumental (monetary)
J. W. Hansen, A. Mishra, K. P. C. Rao, M. Indeje, R. K. Ngugi, Potential value of GCM-based seasonal rainfall forecasts for maize management in semi-arid Kenya. Agricultural Systems 101, 80–90 (2009).	GCM precipitation forecast	Value of information	Agriculture	Instrumental (monetary)
R. Harris, N. Olby, Pricing policy and legal issues: 6th and 7th EOPOLE workshops. Space Policy 16, 287–290 (2000).	various	Market price/cost methods	Various	Instrumental (monetary)
J. Haskins, et al., Uav to inform restoration: a case study from a california tidal marsh. Frontiers In Environmental Science (2021).	UAV	Cost-benefit analysis	Ecological Conservation	Instrumental (monetary); Instrumental (non-monetary)
R. Hautala, et al., 'Benefits of meteorological services in South Eastern Europe' (VTT Technical Research Centre of Finland, 2008).	meteorological and hydrological services	Value of information; Market price/cost methods	Various	Instrumental (monetary)
G. C. Hays, et al., Translating marine animal tracking data into conservation policy and management. Trends In Ecology & Evolution (2019).	marine animal tracking data	Semi-structured and in-depth interviews	Ecological Conservation	Instrumental (non-monetary)
L. Heldt, P. Beske-Janssen, Solutions from space? A dynamic capabilities perspective on the growing use of satellite technology for managing sustainability in multi-tier supply chains. International Journal Of Production Economics (2023).	satellite forest monitoring	Semi-structured and in-depth interviews	Agriculture	Instrumental (non-monetary)
V. Herr, et al., A method for estimating the socioeconomic impact of Earth observations in wildland fire suppression decisions. Int. J. Wildland Fire 29, 282 (2020).	MODIS	Value of information	Disasters; Wildland Fires	Instrumental (monetary)
M. Holopainen, M. Talvitie, Effect of data acquisition accuracy on timing of stand harvests and expected net present value. Silva Fennica (2006).		Value of information; Cost-benefit analysis	Agriculture	Instrumental (monetary)

Reference	ESI source	Valuation method(s)	Decision context	Value type(s)
J. Honey-Roses, J. Lopez-Garcia, E. Rendon-Salinas, A. Peralta-Higuera, C. Galindo-Leal, To pay or not to pay? Monitoring performance and enforcing conditionality when paying for forest conservation in mexico. Environmental Conservation (2009).	aerial imagery	Value of information	Ecological Conservation	Instrumental (monetary); Instrumental (non-monetary)
M. Isik, D. Hudson, K. Coble, The value of site-specific information and the environment: Technology adoption and pesticide use under uncertainty. Journal Of Environmental Management (2005).	remote sensing	Cost-benefit analysis; Real options analysis	Agriculture	Instrumental (monetary)
C. Jabbour, A. Hoayek, P. Maurel, H. Rey-Valette, J.-M. Salles, How much would you pay for a satellite image?: Lessons learned from french spatial-data infrastructure. Ieee Geoscience And Remote Sensing Magazine (2020).	GEOSUD	Stated preference	Various	Instrumental (monetary)
C. Jabbour, A. Hoayek, J.-M. Salles, Formalizing a two-step decision-making process in land use: Evidence from controlling forest clearcutting using spatial information. Land (2023).	GEOSUD	Bayesian decision analysis; Stated preference	Agriculture	Instrumental (monetary)
K. Jantke, C. Schleupner, U. A. Schneider, Benefits of earth observation data for conservation planning in the case of european wetland biodiversity. Environmental Conservation (2013).		Cost-benefit analysis	Ecological Conservation	Instrumental (monetary); Instrumental (non-monetary)
D. Jin, P. Hoagland, The value of harmful algal bloom predictions to the nearshore commercial shellfish fishery in the Gulf of Maine. Harmful Algae 7, 772–781 (2008).	HAB predictions (not necessarily EO based)	Value of information	Agriculture	Instrumental (monetary)
J. W. Jones, J. W. Hansen, F. S. Royce, C. D. Messina, Potential benefits of climate forecasting to agriculture. Agriculture, Ecosystems & Environment 82, 169–184 (2000).	ENSO forecasts	Value of information	Agriculture	Instrumental (monetary)
M. J. Kaiser, A. G. Pulsipher, The potential value of improved ocean observation systems in the Gulf of Mexico. Marine Policy 28, 469–489 (2004).	Ocean observing network	Value of information	Various	Instrumental (monetary)
A. Kangas, T. Gobakken, S. Puliti, M. Hauglin, E. Naesset, Value of airborne laser scanning and digital aerial photogrammetry data in forest decision making. Silva Fennica (2018).	airborne laser scanning; digital aerial photogrammetry	Value of information	Agriculture	Instrumental (monetary)
T. Keenan, et al., The sydney 2000 world weather research programme forecast demonstration project. Bulletin Of The American Meteorological Society (2003).	Nine different observationally based nowcasting systems	Surveys of preference assessments	Climate & Resilience	Instrumental (monetary)
P. L. Kenkel, P. E. Norris, Agricultural Producers' Willingness to Pay for Real-Time Mesoscale Weather Information. Journal of Agricultural and Resource Economics 20, 356–372 (1995).	Mesonet weather network	Stated preference	Agriculture	Instrumental (monetary)
N. Khabarov, E. Moltchanova, M. Obersteiner, Valuing Weather Observation Systems For Forest Fire Management. IEEE Systems Journal 2, 349–357 (2008).	Aerial observation data	Value of information	Disasters; Wildland Fires	Instrumental (monetary)
J.-H. Kim, H. Lim, J. Shin, S.-H. Yoo, Evaluating the public value of improving early detection accuracy of cumulonimbus using a geostationary satellite in south korea. Space Policy (2022).	Cheollian Satellite 2A called Geo-Kompsat-2A	Stated preference	Climate & Resilience	Instrumental (monetary)
H. Kite-Powell, The Value of Ocean Surface Wind Information for Maritime Commerce. mar technol soc j 45, 75–84 (2011).	Various instrument systems	Value of information	Climate & Resilience	Instrumental (monetary)
A. Koppa, et al., A Scalable Earth Observations-Based Decision Support System for Hydropower Planning in Africa. J American Water Resour Assoc 57, 711–736 (2021).	Earth Observing System derived P and ET datasets	Value of information	Water Resources	Instrumental (non-monetary)

Reference	ESI source	Valuation method(s)	Decision context	Value type(s)
S. V. Kumar, K. W. Harrison, C. D. Peters-Lidard, J. A. Santanello, D. Kirschbaum, Assessing the impact of l-band observations on drought and flood risk estimation: a decision-theoretic approach in an osse environment. Journal Of Hydrometeorology (2014).	Simulations based on SMAP Radiometer	Value of information	Agriculture; Water Resources	Instrumental (monetary)
A. L'Astorina, I. Tomasoni, A. Basoni, P. Carrara, Beyond the dissemination of earth observation research: Stakeholders' and users' involvement in project co-design. Journal Of Science Communication (2015).	remote sensing	Econometric analysis	Agriculture	Instrumental (monetary)
J. A. Larson, et al., Factors affecting farmer adoption of remotely sensed imagery for precision management in cotton production. Precision Agriculture (2008).	Earth observation technologies	Semi-structured and in-depth interviews	Agriculture; Capacity Building	Instrumental (monetary)
C. Lauer, J. Conran, J. Adkins, Estimating the Societal Benefits of Satellite Instruments: Application to a Break-even Analysis of the GeoXO Hyperspectral IR Sounder. Frontiers in Environmental Science 9 (2021).	GeoXO Hyperspectral Sounder	Value of information; Surveys of preference assessments	Climate & Resilience	Instrumental (monetary)
J. K. Lazo, L. Chestnut, Economic Value of Current and Improved Weather Forecasts in the U.S. Household Sector. (2002).	NWS weather forecast	Stated preference; Value of information	Climate & Resilience; Various	Instrumental (monetary); Instrumental (non-monetary); Relational
D. Letson, et al., Value of perfect ENSO phase predictions for agriculture: evaluating the impact of land tenure and decision objectives. Climatic Change 97, 145–170 (2009).	ENSO forecasts	Value of information	Agriculture	Instrumental (monetary)
M. Li, A. Faghri, A. Ozden, Y. Yue, Economic feasibility study for pavement monitoring using synthetic aperture radar-based satellite remote sensing cost-benefit analysis. Transportation Research Record (2017).	SAR	Cost-benefit analysis	Other	Instrumental (monetary)
S.-Y. Liao, C.-C. Chen, S.-H. Hsu, Estimating the value of El Niño Southern Oscillation information in a regional water market with implications for water management. Journal of Hydrology 394, 347–356 (2010).	ENSO forecasts	Value of information; Econometric analysis	Water Resources	Instrumental (monetary); Instrumental (non-monetary)
S. H. Lim, Y. Ge, J. M. Jacobs, X. Jia, Measuring the economic benefits of advanced technology use for river flood forecasting. Journal Of Flood Risk Management (2022).	satellite SWE observations	Stated preference; Econometric analysis	Agriculture	Instrumental (monetary)
C. Linés, A. Iglesias, L. Garrote, V. Sotés, M. Werner, Do users benefit from additional information in support of operational drought management decisions in the Ebro basin? Hydrol. Earth Syst. Sci. 22, 5901–5917 (2018).	General remote sensing	Value of information; Real options analysis	Agriculture; Water Resources	Instrumental (monetary)
J. Loomis, S. Koontz, H. Miller, L. Richardson, Valuing Geospatial Information: Using the Contingent Valuation Method to Estimate the Economic Benefits of Landsat Satellite Imagery. Photogrammetric Engineering & Remote Sensing 81, 647–656 (2015).	Landsat	Stated preference	Various	Instrumental (monetary)
W. K. Luseno, J. G. McPeak, C. B. Barrett, P. D. Little, G. Gebru, Assessing the Value of Climate Forecast Information for Pastoralists: Evidence from Southern Ethiopia and Northern Kenya. World Development 31, 1477–1494 (2003).	climate forecasts	Semi-structured and in-depth interviews; Surveys of preference assessments	Agriculture	Instrumental (monetary); Instrumental (non-monetary)
M. K. Macauley, The value of information: Measuring the contribution of space-derived earth science data to resource management. Space Policy 22, 274–282 (2006).	hypothetical	Value of information	Various	Instrumental (monetary)
B. Maxwell, E. Luschei, Justification for site-specific weed management based on ecology and economics. Weed Science (2005).	remote sensing precipitation data	Value of information	Agriculture	Instrumental (monetary)

Reference	ESI source	Valuation method(s)	Decision context	Value type(s)
I. McCallum, et al., Banda Aceh-The Value of Earth Observation Data in Disaster Recovery and Reconstruction: A Case Study. (2008).	earth observation data	Surveys of preference assessments; Cost-benefit analysis	Disasters; Water Resources	Instrumental (monetary)
B. M. Miller, The Not-So-Marginal Value of Weather Warning Systems. Weather, Climate, and Society 10, 89–101 (2018).	weather warning system	Econometric analysis	Climate & Resilience	Instrumental (monetary); Instrumental (non-monetary)
H. M. Miller, L. A. Richardson, S. R. Koontz, J. Loomis, L. Koontz, "Users, uses, and value of Landsat satellite imagery: results from the 2012 survey of users" (U.S. Geological Survey, 2013).	Landsat	Surveys of preference assessments; Stated preference	Various	Instrumental (monetary)
A. Millner, Getting the Most out of Ensemble Forecasts: A Valuation Model Based on User–Forecast Interactions. Journal of Applied Meteorology and Climatology 47, 2561–2571 (2008).	hypothetical weather forecast	Bayesian decision analysis	Climate & Resilience	Instrumental (monetary)
J. Moellmann, M. Buchholz, O. Musshoff, Comparing the hedging effectiveness of weather derivatives based on remotely sensed vegetation health indices and meteorological indices. Weather Climate And Society (2018).	AVHRR	Econometric analysis	Agriculture	Instrumental (monetary)
E. B. Molder, S. F. Schenkein, A. E. McConnell, K. K. Benedict, C. L. Straub, Landsat Data Ecosystem Case Study: Actor Perceptions of the Use and Value of Landsat. Frontiers in Environmental Science 9 (2022).	Landsat	Semi-structured and in-depth interviews	Various	Instrumental (monetary); Instrumental (non-monetary)
E. Moltchanova, N. Khabarov, M. Obersteiner, D. Ehrlich, M. Moula, The value of rapid damage assessment for efficient earthquake response. Safety Science (2011).	hypothetical earthquake rapid response based on earth observation	Value of information; Cost-benefit analysis	Disasters	Instrumental (monetary); Instrumental (non-monetary)
J. Morgenroth, R. Visser, Uptake and barriers to the use of geospatial technologies in forest management. New Zealand Journal Of Forestry Science (2013).	aerial photography, lidar, radar	Surveys of preference assessments	Agriculture	Instrumental (monetary)
V. Morretta, M. Florio, M. Landoni, The social value of earth observation: a new evaluation framework for public high-tech infrastructures. Structural Change And Economic Dynamics (2023).	hypothetical	Cost-benefit analysis	Various	Instrumental (monetary); Instrumental (non-monetary); Relational
V. Morretta, D. Vurchio, S. Carrazza, The socio-economic value of scientific publications: The case of Earth Observation satellites. Technological Forecasting and Social Change 180, 121730 (2022).	Cosmo Skymed	Cost-benefit analysis	Various	Instrumental (monetary); Relational
J. Musinsky, et al., Conservation impacts of a near real-time forest monitoring and alert system for the tropics. Remote Sensing In Ecology And Conservation (2018).	MODIS, VIIRS active fire data	Surveys of preference assessments; Semi-structured and in-depth interviews	Agriculture; Various	Instrumental (monetary); Instrumental (non-monetary)
S. C. Newbold, S. Lindley, S. Albeke, J. Viers, R. Johnston, 'Valuing Satellite Data for Harmful Algal Bloom Early Warning Systems' (Resources for the Future, 2022).	HAB warning system based on satellite imagery	Value of information; Revealed preference	Water Resources	Instrumental (monetary)
N. Nikolic, et al., Site- and time-specific early weed control is able to reduce herbicide use in maize - a case study. Italian Journal Of Agronomy (2021).	UAV	Value of information	Agriculture	Instrumental (monetary)
L. Noordermeer, T. Gobakken, E. Naesset, O. M. Bollandsas, Economic utility of 3d remote sensing data for estimation of site index in nordic commercial forest inventories: a comparison of airborne laser scanning, digital aerial photogrammetry and conventional practices. Scandinavian Journal Of Forest Research (2021).	Airborne laser scanning and digital aerial photogrammetry	Value of information; Cost-benefit analysis	Agriculture	Instrumental (monetary)

Reference	ESI source	Valuation method(s)	Decision context	Value type(s)
F. Nutini, et al., Supporting operational site-specific fertilization in rice cropping systems with infield smartphone measurements and sentinel-2 observations. Precision Agriculture (2021).	Sentinel	Value of information	Agriculture	Instrumental (monetary)
K. O'Dell, et al., Public health benefits from improved identification of severe air pollution events with geostationary satellite data. (2023).	GEOSS	Value of information; Cost-benefit analysis	Various	Instrumental (monetary)
M. Obersteiner, F. Rydzak, S. Fritz, I. McCallum, Valuing the potential impacts of geoss: a systems dynamics approach. The Value Of Information: Methodological Frontiers And New Applications In Environment And Health (2012).	MODIS	Value of information	Disasters	Instrumental (monetary)
P. C. Oddo, J. D. Bolten, The Value of Near Real-Time Earth Observations for Improved Flood Disaster Response. Frontiers in Environmental Science 7 (2019).	GOES;VIIRS;	Value of information	Health & Air Quality	Instrumental (monetary); Instrumental (non-monetary)
R. Opitz, et al., Practicing critical zone observation in agricultural landscapes: Communities, technology, environment and archaeology. Land (2023).	various	Semi-structured and in-depth interviews; Focus groups	Agriculture; Capacity Building	Instrumental (monetary)
B. P. Parajuli, et al., An open data and citizen science approach to building resilience to natural hazards in a data-scarce remote mountainous part of nepal. Sustainability (2020).	Satellite imagery	Non-monetary methods - deliberative	Various; Capacity Building	Instrumental (non-monetary); Relational
S.-Y. Park, S.-H. Yoo, The public value of improving a weather forecasting system in Korea: a choice experiment study. Applied Economics 50, 1644–1658 (2018).	weather forecast	Stated preference	Climate & Resilience	Instrumental (monetary)
F. Pearlman, R. Bernknopf, M. A. Stewart, J. S. Pearlman, Impacts of geospatial information for decision making. Advances In Natural And Technological Hazards Research (2014).	MRLI (Landsat); PRISM	Value of information; Cost-benefit analysis	Health & Air Quality; Agriculture	Instrumental (monetary)
E. H. Petersen, R. W. Fraser, An assessment of the value of seasonal forecasting technology for Western Australian farmers. Agricultural Systems 70, 259–274 (2001).	climate forecasts	Value of information	Agriculture	Instrumental (monetary)
S. Quiroga, et al., The economic value of drought information for water management under climate change: a case study in the Ebro basin. Nat. Hazards Earth Syst. Sci. 11, 643–657 (2011).	drought forecast	Value of information	Agriculture	Instrumental (monetary)
A. Rango, Operational applications of satellite snow cover observations. Jawra Journal Of The American Water Resources Association (1980).	Landsat, VHRR	Cost-benefit analysis	Water Resources	Instrumental (monetary)
R. D. Roberts, et al., Taking the highway to save lives on lake victoria. Bulletin Of The American Meteorological Society (2022).	weather warning system	Focus groups; Semi-structured and in-depth interviews	Climate & Resilience	Instrumental (monetary); Instrumental (non-monetary)
K. S. Rollins, J. Shaykewich, Using willingness-to-pay to assess the economic value of weather forecasts for multiple commercial sectors. Meteorological Applications 10, 31–38 (2003).	weather forecast	Stated preference	Climate & Resilience	Instrumental (monetary)
K. W. Ross, M. E. Brown, J. P. Verdin, L. W. Underwood, Review of fews net biophysical monitoring requirements. Environmental Research Letters (2009).	FEWS NET	Surveys of preference assessments	Agriculture; Climate & Resilience	Instrumental (monetary)
T. F. Rotheli, Applied welfare economics with bounded rationality: Public policies toward remote sensing. International Advances In Economic Research (2005).	hypothetical crop health	Cost-benefit analysis	Agriculture	Instrumental (monetary)
M. Rouget, Measuring conservation value at fine and broad scales: Implications for a diverse and fragmented region, the agulhas plain. Biological Conservation (2003).	remote sensing at different scales	Value of information	Ecological Conservation	Instrumental (monetary); Instrumental (non-monetary)

Reference	ESI source	Valuation method(s)	Decision context	Value type(s)
F. Rydzak, M. Obersteiner, F. Kraxner, Impact of Global Earth Observation - Systemic view across GEOSS societal benefit areas. International Journal of Spatial Data Infrastructures Research 216–243 (2010).	GEOSS	Value of information	Various	Instrumental (monetary); Instrumental (non-monetary)
V. Šafář, et al., The role of remote sensing in agriculture and future vision. Agris On-Line Papers In Economics And Informatics (2022).	Copernicus	Surveys of preference assessments; Focus groups	Agriculture	Instrumental (monetary)
V. G. Sales, E. Strobl, R. J. R. Elliott, Cloud cover and its impact on brazil's deforestation satellite monitoring program: Evidence from the cerrado biome of the brazilian legal amazon. Applied Geography (2022).	multispectral remote radar	Value of information	Climate & Resilience; Agriculture	Instrumental (monetary); Instrumental (non-monetary)
P. G. Sassone, The economics of atmosphere monitoring systems: Theory and applications. Climatic Change (1982).	atmosphere monitoring systems	Value of information	Health & Air Quality	Instrumental (monetary)
G. Sawyer, E. Mamais, D. Papadakis, The Six Dimensions of Value Associated to the use of Copernicus Sentinel Data: Key Findings From the Sentinel Benefits Study. Frontiers in Environmental Science 10 (2022).	Sentinel	Value of information	Various	Instrumental (monetary); Instrumental (non-monetary); Relational
E. Schiavon, et al., Maximizing societal benefit across multiple hyperspectral earth observation missions: a user needs approach. Journal Of Geophysical Research-Biogeosciences (2023).		Focus groups; Semi-structured and in-depth interviews	Various	Instrumental (monetary); Instrumental (non-monetary)
C. Schweik, C. Thomas, Using remote sensing to evaluate environmental institutional designs: a habitat conservation planning example. Social Science Quarterly (2002).	LandSat	Cost-benefit analysis	Ecological Conservation	Instrumental (non-monetary)
S. Seelan, S. Laguette, G. Casady, G. Seielstad, Remote sensing applications for precision agriculture: a learning community approach. Remote Sensing Of Environment (2003).	AVHRR, MODIS, ETM+, IKONOS, Digit Inc's DALSA camera system and Positive Systems' ADAR 5500 digital aerial camera	Non-monetary methods - deliberative	Agriculture; Capacity Building	Instrumental (monetary); Instrumental (non-monetary); Relational
G. A. Seielstad, et al., Applications of remote sensing to precision agriculture with dual economic and environmental benefits. Proceedings Of Spie-The International Society For Optical Engineering (2002).	AVHRR; ETM+; IKONOS; ADAR5500; MODIS	Value of information; Non-monetary methods - deliberative	Agriculture	Instrumental (monetary)
J. C. Selgrath, C. Roelfsema, S. E. Gergel, A. C. J. Vincent, Mapping for coral reef conservation: Comparing the value of participatory and remote sensing approaches. Ecosphere (2016).	Digital Globe Worldview 2	Value of information; Cost-benefit analysis	Ecological Conservation	Instrumental (non-monetary)
V. Sharda, P. Srivastava, Value of ENSO-Forecasted Drought Information for the Management of Water Resources of Small to Mid-Size Communities. Transactions of the ASABE (American Society of Agricultural and Biological Engineers) 59, 1733–1744 (2016).	ENSO forecasts	Value of information	Water Resources	Instrumental (monetary); Instrumental (non-monetary)
K. Smith, R. Berry, L. E. Clarke, Exploring the potential of google earth as a communication and engagement tool in collaborative natural flood management planning. Geographical Journal (2020).	Google Earth	Focus groups; Surveys of preference assessments	Disasters; Water Resources	Instrumental (monetary); Instrumental (non-monetary); Relational
I. S. Smythe, J. E. Blumenstock, Geographic microtargeting of social assistance with high-resolution poverty maps. Proc. Natl. Acad. Sci. U.S.A. 119, e2120025119 (2022).	satellite imagery	Value of information	Capacity Building	Instrumental (monetary); Relational
A. R. Solow, et al., The Value of Improved ENSO Prediction to U.S. Agriculture. Climatic Change 39: 47–60 (1998).	ENSO forecasts	Bayesian decision analysis	Agriculture	Instrumental (monetary)

Reference	ESI source	Valuation method(s)	Decision context	Value type(s)
M. Sozzi, et al., Economic comparison of satellite, plane and uav-acquired ndvi images for site-specific nitrogen application: Observations from italy. Agronomy-Basel (2021).	Satellite imagery, aerial imagery, UAV	Value of information; Cost-benefit analysis	Agriculture	Instrumental (monetary)
K. Spaeti, R. Huber, R. Finger, Benefits of increasing information accuracy in variable rate technologies. Ecological Economics (2021).	satellite imagery, drone imagery	Value of information; Cost-benefit analysis	Agriculture	Instrumental (monetary)
J. H. Stel, B. F. Mannix, A benefit-cost analysis of a regional global ocean observing system: Seawatch Europe. Marine Policy 20, 357–376 (1996).	Seawatch system	Cost-benefit analysis	Various	Instrumental (monetary)
S. Stroming, M. Robertson, B. Mabee, Y. Kuwayama, B. Schaeffer, Quantifying the Human Health Benefits of Using Satellite Information to Detect Cyanobacterial Harmful Algal Blooms and Manage Recreational Advisories in U.S. Lakes. Geohealth 4, e2020GH000254 (2020).	Sentinel-3	Value of information; Market price/cost methods	Water Resources; Health & Air Quality	Instrumental (monetary); Instrumental (non-monetary)
D. M. Styers, Using big data to engage undergraduate students in authentic science. Journal Of Geoscience Education (2018).	MODIS; Landsat	Surveys of preference assessments	Various; Capacity Building	Relational
D. M. Sullivan, A. Krupnick, Using Satellite Data to Fill the Gaps in the US Air Pollution Monitoring Network. (2019).	various satellite	Value of information; Econometric analysis	Health & Air Quality	Instrumental (non-monetary)
Y. Tang, et al., Grid-scale agricultural land and water management: a remote-sensing-based multiobjective approach. Journal Of Cleaner Production (2020).	MODIS	Value of information	Agriculture; Water Resources	Instrumental (monetary); Instrumental (non-monetary)
T. Tanhuanpaa, et al., Input data resolution affects the conservation prioritization outcome of spatially sparse biodiversity features. Ambio (2023).	Simulated data at various resolutions	Value of information	Ecological Conservation	Instrumental (non-monetary)
A. Taramelli, et al., An interaction methodology to collect and assess user-driven requirements to define potential opportunities of future hyperspectral imaging sentinel mission. Remote Sensing (2020).	Sentinel	Surveys of preference assessments; Delphi method	Various	Instrumental (monetary)
A. Tassa, S. Willekens, A. Lahcen, L. Laurich, C. Mathieu, On-Going European Space Agency Activities on Measuring the Benefits of Earth Observations to Society: Challenges, Achievements and Next Steps. Frontiers in Environmental Science 10 (2022).	ESA missions	Value of information	Various	Instrumental (monetary)
W. Toombs, et al., Use and benefits of nasa's recover for post-fire decision support. International Journal Of Wildland Fire (2018).	RECOVER post-fire decision support system	Semi-structured and in-depth interviews	Wildland Fires	Instrumental (monetary); Instrumental (non-monetary)
S. N. Trigg, D. P. Roy, A focus group study of factors that promote and constrain the use of satellite-derived fire products by resource managers in southern africa. Journal Of Environmental Management (2007).	MODIS	Focus groups; Semi-structured and in-depth interviews	Wildland Fires; Capacity Building	Instrumental (non-monetary)
K. R. Varshney, et al., Targeting villages for rural development using satellite image analysis. Big Data (2015).	satellite imagery	Cost-benefit analysis	Capacity Building	Instrumental (monetary); Instrumental (non-monetary); Relational
F. Vuolo, L. Essl, C. Atzberger, Costs and benefits of satellite-based tools for irrigation management. Frontiers In Environmental Science (2015).	Landsat; DEIMOS	Cost-benefit analysis; Semi-structured and in-depth interviews	Agriculture; Water Resources	Instrumental (monetary)
H. Wang, et al., Drone-based harvest data prediction can reduce on-farm food loss and improve farmer income. Plant Phenomics (2023).	drone	Value of information	Agriculture	Instrumental (monetary); Instrumental (non-monetary)

Reference	ESI source	Valuation method(s)	Decision context	Value type(s)
K. F. Wellman, M. Hartley, Potential Benefits of Coastal Ocean Observing Systems to Alaskan Commercial Fisheries. Coastal Management 36, 193–207 (2008).	Alaska Ocean Observing System	Value of information	Agriculture	Instrumental (monetary); Instrumental (non-monetary)
K. Wieand, A Bayesian Methodology for Estimating the Impacts of Improved Coastal Ocean Information on the Marine Recreational Fishing Industry. Coastal Management 36, 208–223 (2008).	Integrated Ocean Observation System	Bayesian decision analysis	Agriculture	Instrumental (monetary)
S. Wikberg, et al., Cost-effectiveness of conservation strategies implemented in boreal forests: The area selection process. Biological Conservation (2009).	satellite imagery	Cost-benefit analysis; Value of information	Ecological Conservation	Instrumental (monetary)
D. S. Wilks, A skill score based on economic value for probability forecasts. Meteorological Applications 8, 209–219 (2001).	hypothetical weather forecast	Value of information	Climate & Resilience	Instrumental (monetary)
C. Yeh, et al., Using publicly available satellite imagery and deep learning to understand economic well-being in africa. Nature Communications (2020).	Landsat; night light data	Value of information	Capacity Building	Instrumental (monetary)
D. R. Zeh, et al., Is acoustic tracking appropriate for air-breathing marine animals? Dugongs as a case study. Journal Of Experimental Marine Biology And Ecology (2015).	satellite and acoustic telemetry	Cost-benefit analysis	Ecological Conservation	Instrumental (monetary); Instrumental (non-monetary)
J. R. Ziolkowska, Economic value of environmental and weather information for agricultural decisions - A case study for Oklahoma Mesonet. Agriculture, Ecosystems & Environment 265, 503–512 (2018).	Mesonet weather network	Value of information	Agriculture	Instrumental (monetary)

PNAS

Papers by method, decision context, value type

Table S4. Papers included by method, decision context, and value type. The top number in each cell represents the total number of documents (out of 171) that include that combination of method and societal benefit decision context. The bottom three numbers indicate the number of those papers that examine monetary instrumental, non-monetary instrumental, and relational value types, respectively.

	Societal benefit decision context									
Valuation method	Agriculture	Various	Climate & Resilience	Water Resources	Ecological Conservation	Capacity Building	Disasters	Health & Air Quality	Wildland Fires	Other
**Value of information**	**44**<br>43 - 9 - 1	**9**<br>9 - 4 - 2	**11**<br>10 - 5 - 2	**16**<br>14 - 6 - 0	**8**<br>6 - 5 - 0	**3**<br>3 - 1 - 1	**4**<br>4 - 1 - 0	**6**<br>5 - 4 - 0	**2**<br>2 - 0 - 0	
**Cost-benefit analysis**	**10**<br>10 - 2 - 0	**7**<br>7 - 2 - 2	**2**<br>2 - 1 - 0	**4**<br>4 - 0 - 0	**9**<br>7 - 6 - 0	**1**<br>1 - 1 - 1	**3**<br>3 - 1 - 0	**1**<br>1 - 0 - 0	**1**<br>1 - 0 - 0	**1**<br>1 - 0 - 0
**Surveys of preference assessments**	**12**<br>12 - 6 - 1	**5**<br>4 - 2 - 2	**4**<br>4 - 1 - 0	**2**<br>2 - 1 - 1	**5**<br>5 - 4 - 1	**4**<br>3 - 2 - 2	**3**<br>3 - 1 - 1		**1**<br>1 - 0 - 0	**1**<br>0 - 1 - 1
**Semi-structured and in-depth interviews**	**14**<br>11 - 10 - 3	**5**<br>4 - 5 - 2	**2**<br>1 - 2 - 0	**1**<br>1 - 0 - 0	**2**<br>1 - 2 - 1	**7**<br>4 - 4 - 3			**2**<br>1 - 2 - 0	
**Stated preference**	**5**<br>5 - 0 - 0	**4**<br>4 - 1 - 1	**4**<br>4 - 1 - 1	**1**<br>1 - 0 - 0			**1**<br>1 - 0 - 0	**1**<br>1 - 1 - 0	**1**<br>1 - 0 - 0	
**Bayesian decision analysis**	**8**<br>8 - 1 - 0	**1**<br>1 - 0 - 0	**3**<br>3 - 0 - 0	**1**<br>1 - 0 - 0	**5**<br>5 - 3 - 0					
**Focus groups**	**5**<br>4 - 2 - 1	**2**<br>1 - 2 - 1	**1**<br>1 - 1 - 0	**1**<br>1 - 1 - 1		**4**<br>1 - 3 - 2	**1**<br>1 - 1 - 1		**1**<br>0 - 1 - 0	
**Econometric analysis**	**6**<br>6 - 0 - 0		**2**<br>2 - 1 - 0	**2**<br>2 - 1 - 0				**2**<br>0 - 2 - 0		
**Real options analysis**	**3**<br>3 - 0 - 0		**1**<br>1 - 0 - 0	**1**<br>1 - 0 - 0			**1**<br>1 - 0 - 0			
**Non-monetary methods - deliberative**	**2**<br>2 - 1 - 1	**1**<br>0 - 1 - 1				**2**<br>1 - 2 - 2				
**Market price/cost methods**		**2**<br>2 - 0 - 0		**1**<br>1 - 1 - 0				**1**<br>1 - 1 - 0		
**Revealed preference**				**1**<br>1 - 0 - 0						
**Delphi method**		**1**<br>1 - 0 - 0								

Methods

Search string

Consolidated search term (January 26, 2024) included several broad topics: Earth science information; a decision context or value analysis; and some notion of societal benefit. Each of these broad topics was encoded as a collection of related terms joined by OR logic to maximize inclusivity within the topic; then the three topics were joined using AND logic to identify papers at the intersection of the three broad topics.

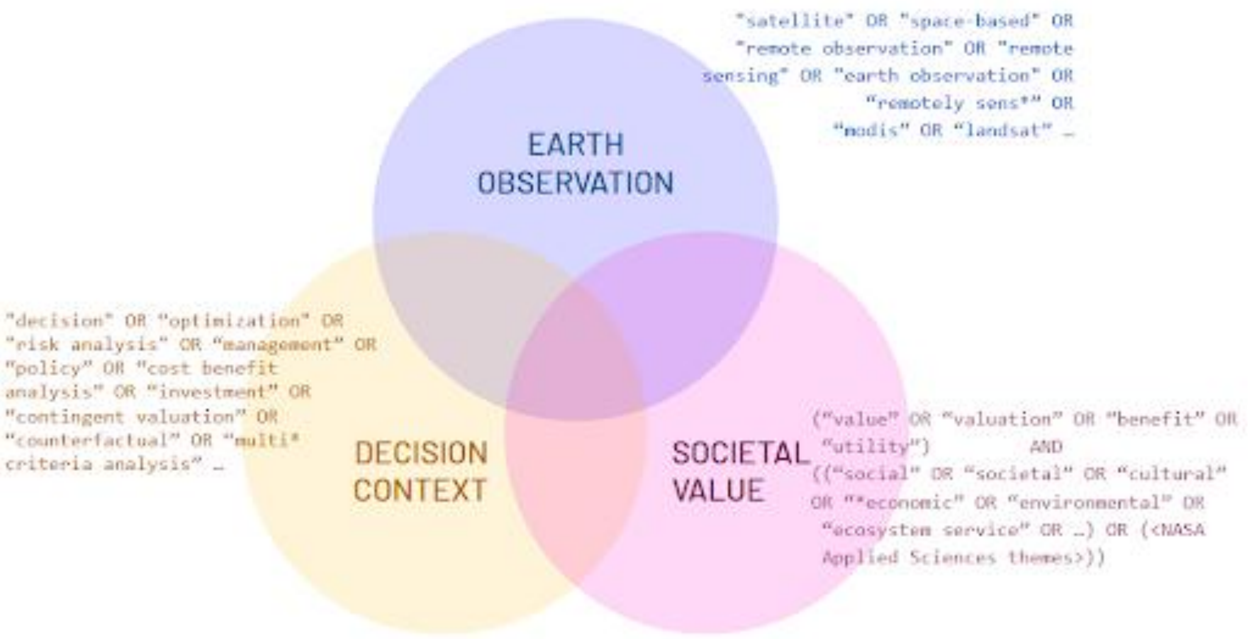

Figure S1. Conceptual diagram of search string.

Terms in italics are from the exploratory search on October 25, 2023; terms in bold were added following the use of the litsearchr R package by Grames et al. (3); terms in bold italics were added following discussion at the American Geophysical Union conference in December 2023. The final Web of Science search was performed using these search strings on January 26, 2024; the final Scopus search was performed using these search strings on February 4, 2024.

- Earth science information terms:
  - (*"satellite" OR "space-based" OR "remote observation" OR "remote sensing" OR "earth observation"* OR **"remotely sens*" OR "MODIS" OR "Landsat"** OR ***"GRACE" OR "SRTM" OR "Sentinel" OR "VIIRS" OR "TERRA" OR "CLARREO"***)

- Decision context terms:
  - (*"decision" OR "optimization" OR "risk analysis" OR "management" OR "policy"* OR **"cost benefit analysis" OR "benefit cost analysis" OR "investment" OR "contingent valuation" OR "counterfactual"** OR ***"value chain analysis" OR "multi* criteria analysis" OR "multi* criteria decision analysis" OR "planning" OR "governance" OR***

"prioritization" OR "impact assessment" OR "impact evaluation" OR "willingness to pay")

- Societal benefit terms:
  - (*"value*" OR "valuation" OR "benefit*"* OR **"utility"**) AND (*"social" OR "societal" OR "cultural" OR "*economic"* OR **"environmental" OR "ecosystem service" OR "sustainable development" OR "protected area" OR "heritage site" OR "non use value" OR "capacity building" OR "disaster" OR "water resource*" OR "climate resilience" OR "air quality" OR "conservation" OR "wildland fire*" OR "wildfire"** OR ***"empower*" OR "power structure*" OR "justice" OR "equit*" OR "financial" OR "monetary" OR "health" OR "well-being" OR "livelihood" OR "community-*" OR "inspiration*" OR "educat*" OR "arts" OR "familial" OR "spiritual" OR "religious"***)

Screening process

Preliminary screening of spurious matches

An early examination of search results showed that many of the ESI-focused terms resulted in spurious matches, since many of those terms on their own have alternate meanings unrelated to ESI. For example, "satellite" is used to describe sub-nodes in networks such as libraries or medical clinics; in medical research, "sentinel" (relating to the ESA's Copernicus mission) can refer to lymph nodes and cells observed for early detection of cancers; and "terra" (relating to one of two satellites equipped with MODIS sensors) can be paired with "preta" to describe the carbon-rich black soil found in indigenous regions of the Amazon. To eliminate some of the most common instances of these spurious matches, we identified a set of terms to be excluded using regular expressions for flexibility; if these terms were removed from titles/abstracts and no other terms in the title or abstract matched other ESI-related terms, then that document would be excluded from further consideration.

- "Satellite" terms:
  - 'satellite' plus any of: 'account', 'office', 'laborator(y|ies)', 'campus', '([a-z]+.)?clinic', '([a-z]+.)?hospital', '([a-z]+.)?cent(er|re)', 'lesion', 'nodule', 'mass', 'h(a)?emodialysis'
- "Sentinel" terms (relating to the Sentinel satellites of ESA's Copernicus programme):
  - 'sentinel' plus any of: 'study', '(lymph.)?node', 'site', '([a-z]+.)?surveillance', 'species', 'behavior', 'catalyst', 'event'
- "Grace" terms (relating to NASA/JPL Gravity Recovery and Climate Experiment mission):
  - 'grace.period'
- "Terra" terms (relating to NASA's Terra MODIS satellite):
  - 'Terra' plus one of: 'preta', 'nova', 'firme', 'nullius'
- Health terms that frequently showed up in spurious matches:
  - Any of 'cancer', 'cardiac', 'cardio'

Screening criteria

Exclusion criteria used in the citation screening (title + abstract) and full text phases:

- ESI data are not used:
  - No relation to Earth science information. For example, spurious matches related to health care remote observation.

  - Related to satellites but not related to information about Earth's systems. For example, documents relating to space weather, solar or lunar information, or communications/navigation satellites.
- Data are not valued
  - ESI data are used to determine some scientific finding, but the scientific finding is not used to inform a specific societal decision or otherwise valued.
  - For example, ESI data used to estimate changes in ecosystem service value over time, but the resulting ecosystem service value is not used to inform any management decisions within the paper - i.e., the ESI measurement did not generate value.
- Valued data is not ESI
  - Valuation methods are used in the paper, but applied to data or information other than the ESI. For example, a study that applies a new classification algorithm to the same underlying data; in this case, the additional value is attributable to the algorithm rather than the underlying data.
- Review/opinion
  - Document is a review or opinion piece and does not provide new analysis or new frameworks for valuation.
- Conference abstract/proceedings
  - Document is a conference abstract or proceeding describing presentations rather than published work
- Validation/calibration
  - A special case of "Data is not valued" - ESI data are used to generate scientific information, and this information is compared to some reference to demonstrate scientific value; however, this scientific value is not then translated into societal benefit.
  - For example, NDVI data is used to estimate land cover, and this result is compared to some alternate information source and shown to be an adequate or even superior proxy, i.e., scientific merit. However, the resulting information is not used to inform a management decision that would translate to some societal benefit.

PNAS

References

1. A. Himes, *et al.*, Why nature matters: A systematic review of intrinsic, instrumental, and relational values. *BioScience* **74**, 25–43 (2024).

2. P. Arias-Arévalo, E. Gómez-Baggethun, B. Martín-López, M. Pérez-Rincón, Widening the Evaluative Space for Ecosystem Services: A Taxonomy of Plural Values and Valuation Methods. *Environmental Values* **27**, 29–53 (2018).

3. E. M. Grames, A. N. Stillman, M. W. Tingley, C. S. Elphick, An automated approach to identifying search terms for systematic reviews using keyword co-occurrence networks. *Methods in Ecology and Evolution* **10**, 1645–1654 (2019).
